# Perspective on the use of fluorescence molecular imaging for peripheral and deep *en face* margin assessment

**DOI:** 10.1117/1.JBO.30.S1.S13711

**Published:** 2025-05-02

**Authors:** Hang M. Nguyen, Veronica C. Torres, Joshua Levy, Eunice Y. Chen, Matthew LeBoeuf, Kimberley S. Samkoe

**Affiliations:** a Dartmouth College, Thayer School of Engineering, Hanover, New Hampshire, United States; b Cedars-Sinai Medical Center, Department of Pathology and Laboratory Medicine, Los Angeles, California, United States; c Dartmouth-Hitchcock Medical Center, Department of Surgery, Lebanon, New Hampshire, United States; d Dartmouth-Hitchcock Medical Center, Department of Dermatology, Lebanon, New Hampshire, United States

**Keywords:** fluorescence, molecular imaging, tumor margin, peripheral and deep *en face* margin assessment

## Abstract

**Significance:**

Current standard practice for margin assessment in solid tumor resection often leads to suboptimal results due to the inability to assess margins completely in a time-efficient manner. On the other hand, for small skin cancers, peripheral and deep *en face* margin assessment (PDEMA) offers 100% assessment of margins while sparing the utmost amount of normal surrounding tissues. Nonetheless, PDEMA is limited in its use owing to its lengthy tissue processing and imaging time as well as its requirement for high-quality frozen sections and real-time histologic analysis.

**Aim:**

We aim to explore fluorescence molecular imaging (FMI) as a tool for resolving obstacles and integrating PDEMA into the surgeon-to-pathologist workflow for large solid tumors.

**Approach:**

A review of recent pre-clinical and clinical studies using FMI to assess surgical margins was conducted to highlight promising fluorescence imaging technologies utilized in the surgical suite and laboratory.

**Results:**

FMI techniques that provide macroscopic resolution are efficient in time and have a notable ability to identify true negative tissue yet have limited capability in identifying true positive tissues. Moreover, meso- and microscopic FMI methods require additional time to attain a higher resolution but deliver an enhanced sensitivity in detecting true positive tissues. In both cases, experts are still required to learn to interpret the FMI signals, which prohibits a seamless clinical integration.

**Conclusions:**

Our proposed margin assessment platform (MAP) incorporates both macroscopic and, meso- or microscopic imaging with post-processing and machine learning for interpretation, to enable the application of PDEMA into solid tumor surgery. MAP leverages the advantages of each technique and thoroughly tackles the limitations of time and expertise to optimize the efficiency and accuracy of margin assessment and ultimately improve clinical outcomes

## 1 Current State of Clinical Margin Assessment

Obtaining tumor-free surgical margins for the treatment of solid tumors is critical to delivering optimal patient outcomes. Positive surgical margins occur either as histologically detected tumor at the edge of a resection specimen (true positive) or a tumor at the edge of a resection specimen not assessed by histologic sampling (false negative). Both true positive margins and false negative margins negatively affect a cancer patient’s prognosis, potentially necessitating additional surgical procedures or adjuvant treatment, including radiation and/or chemotherapy. In addition, both true positive and false negative margins increase morbidity and mortality, as well as monetary and resource costs to the healthcare system. An innovative method to evaluate for and thereby eliminate true positive margins and false negative margins during surgical extirpation of solid tumors will improve the value of surgical cancer treatment and the clinical outcomes of patients.

**Fig. 1:**
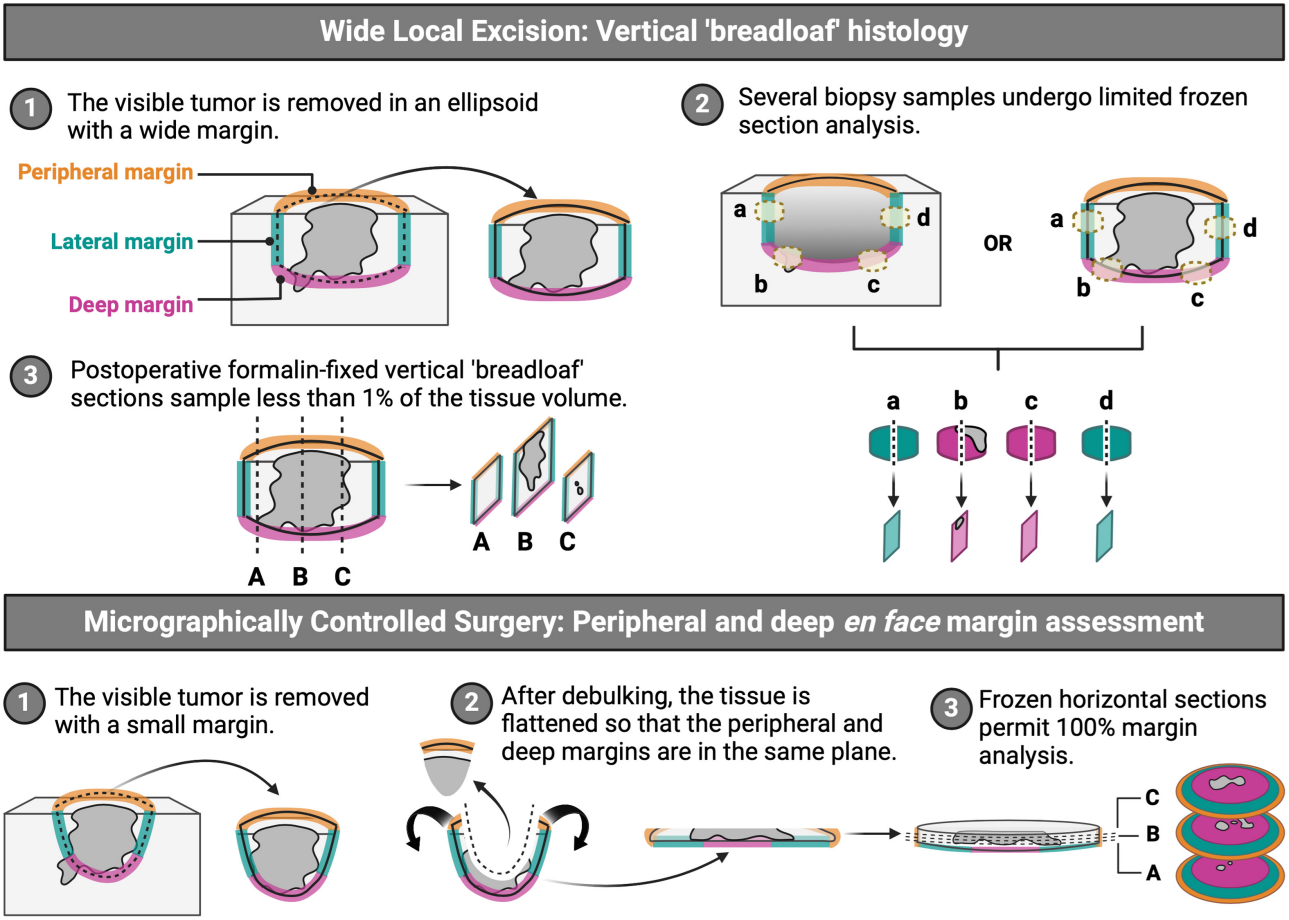
Comparison between breadloaf histology used in conventional wide local excision and peripheral and deep *en face* margin assessment (PDEMA) employed in micrographically controlled surgery. Created with BioRender.

The current surgical workflow for the majority of solid tumors involves intraoperative tumor resection including a 5- to 10-mm normal tissue cuff with orientation suture(s), surgical site closure with or without reconstruction, and tissue fixation for postoperative histopathological diagnosis. Although frozen sections may be taken from the margin to histologically confirm suspicious regions during surgery, official pathological diagnosis and margin determination are performed on the fixed surgical specimen. Processing of the surgical specimen consists of breadloaf or radial sectioning, histologic staining of one tissue slice per section, and pathological analysis in the days to weeks following the completion of the procedure (Fig. 1). Challenges to this current workflow include (1) delayed analysis of the tissue specimens in the event of a positive margin, (2) breadloaf/radial sectioning only assessing ∼1% of the total surgical margin resulting in a false negative surgical margin, and (3) accurately mapping positive tumor margins back to the 3D anatomic surgical space. A recent review of 6,495,889 patients determined that the average rate of positive surgical margins detected after primary resection for the 10 most common solid tumors was 8.85% (range 4.32% to 35.00%), including 12.75% of cases for oral cancers.^1^ However, these numbers do not take into account tumor recurrences that might result from false negative margins at the time of initial margin analysis which occur, on average, in 8% to 24% of cutaneous and oral squamous cell carcinomas.^2^ Taken together, this suggests that ∼25% of all surgeries for these solid tumors result in suboptimal patient outcomes.

In comparison, peripheral and deep *en face* margin assessment (PDEMA) is a technique that allows 100% of the tumor margin to be examined for residual tumor (Fig. 1). The surgical removal of high-risk, non-melanoma skin cancers (NMSC) is most commonly performed using the Mohs Micrographic Surgery (MMS) technique, where entire specimen margins are histologically assessed in real-time using frozen tissue sections, as compared with the Tuebigen torte or muffin techniques that utilize permanent sections. MMS, Tuebigen torte, or muffin techniques are the most common forms of PDEMA; however, in 2022, the National Comprehensive Cancer Network (NCCN) recommended using PDEMA as an encompassing term for whole margin assessment to avoid specialty-specific jargon.^3^ When applied to cutaneous squamous cell carcinomas (CSCC), PDEMA has been shown to have cure rates of 98.1% for tumors <2  cm.^3^ Importantly, in this process, the surgeon resects the tumor carefully, demarcating 12, 3, 6, and 9 o’clock on both the surgical defect and resection specimen, grosses the tissue into an appropriate number of pieces to ensure that (a) peripheral and deep margins are relaxed into the same plane, and (b) the tissue will fit into a tissue block and applies to mark ink that correlates with the 3D location on the original surgical defect. The tissue is handed off to a histotechnician who sections and stains the resulting tissue sections. Once returned to the surgeon, real-time histologic analyses of the tissue sections are performed to detect tumors present at the tissue margins and the ink colors on the tissue sections are used to map the peripheral and deep margins back to the original specimen in 3D space. This iterative process results in (1) complete tumor removal prior to reconstruction of the surgical defect, thereby eliminating the possibility of postoperative positive margins; (2) recurrence rates of ≤1% to 3%;^4^^,^^5^ and (3) sparing of normal surrounding tissue as initial tumor resection margins can be smaller in the setting of the iterative nature of the process. However, although PDEMA provides high-resolution histologic examination for high-risk skin cancers in sensitive areas, it is performed under local anesthesia in a specialized procedural unit and requires a significant investment in time, resources, and expertise to allow for tissue processing, sectioning, histologic analysis, and tumor mapping in an efficient and reliable manner. Due to these substantial requirements, PDEMA is not feasible or practical for larger/deeper solid tumors in the traditional operating room setting under general anesthesia. Significant logistical barriers of the process include (1) time of tissue processing for large tissue specimens, particularly relevant to procedures being performed under general anesthesia; (2) an on-site laboratory capable of producing high-quality frozen tissue sections; and (3) an individual able and capable of performing histologic analysis of frozen sections and tumor mapping in real-time. Although technologies have been proposed to address some of these barriers in the surgical workflow process, the length of time required for tissue processing of large or complex 3D surgical resection specimens remains a limiting factor.^6^

Medical imaging has been demonstrated as a solution to address the above logistical barriers to real-time surgical margin analysis aimed at complete and efficient tumor removal.^7^^,^^8^ Imaging techniques including magnetic resonance imaging (MRI),^9^^–^^11^ ultrasound,^12^^,^^13^ radiography,^14^^,^^15^ computed tomography (CT),^16^^,^^17^ fluorescence-guided surgery (FGS),^18^^,^^19^ and optical coherence tomography (OCT),^20^ as well as advanced microscopy techniques such as confocal mosaicking microscopy,^21^ microscopy with ultraviolet surface excitation (MUSE),^22^ reflectance hyperspectral imaging,^23^^,^^24^ and light sheet microscopy^25^ have been investigated as methods to analyze margins to improve surgical outcomes. However, challenges to both *in situ* and *ex vivo* imaging techniques still remain for balancing imaging time with depth of image and resolution, as well as obtaining the level of expertise required by the surgeon or radiologist to accurately and efficiently analyze the images. A thorough literature review by Heidkamp et al. (2019) was undertaken to assess margin imaging technologies between 2012 and 2018.^7^ This review identified ultrasound, radiography, and fluorescence imaging technologies as the highest in feasibility, with fluorescence being the least developed. However, over the last 3 years, two fluorescence molecular imaging agents (Cytalux and LUMISIGHT) were FDA-approved for three surgical interventions, and >500 manuscripts/year were published on fluorescence-guided surgery. In addition, there are 46 actively recruiting “Fluorescence-Guided Surgery” clinical trials listed on ClinicalTrials.gov, indicating a strong surge of clinical relevance and the development of more advanced detection methodologies.

Within this perspective review, we will examine the idea that fluorescence molecular imaging (FMI) has the potential to overcome the time, expertise, and resolution for assessing whole solid tumor margins, making real-time PDEMA possible in solid tumors that otherwise would not qualify for traditional pathology-based PDEMA. Following the typical PDEMA workflow (Fig. 1), we will discuss common current clinical and pre-clinical FMI methodologies for assessing margins at the macro-, meso-, and microscales and evaluate the advantages and disadvantages of each technique. Current efforts, future directions, and gaps in technology for the integration of PDEMA for solid tumor resection throughout the surgery-to-pathology workflow will be elaborated. Above all, we will present a complete workflow, namely, a margin assessment platform (MAP) that illustrates the incorporation of FMI into each imaging stage to facilitate the application of PDEMA and extend its benefit of 100% margin assessment to solid tumors.

## 2 Current State of FMI for Guided Surgery

Fluorescence imaging for diagnostic surgical guidance has been used for decades, but prior to 2017, the field was limited to perfusion-based imaging agents such as indocyanine green (ICG), fluorescein, and methylene blue. These agents are most commonly used as vascular pooling agents to image perfusion, lymphatic tissues, necrosis, and inflammation. In the past decade, the FDA has approved several other fluorescent agents (Gleolan, Cytalux, LUMISIGHT) that are specific to particular tissue types and are used for identification and resection of tumor tissue that would be otherwise missed by the surgeon *in situ* rather than specifically guiding the identification of pathological tumor margins. Several in-depth review articles on fluorescence molecular-targeted–guided surgery are available that eloquently describe the current state of imaging agents and targets that are beyond the scope of this perspective review.^26^^–^^28^ In addition, relative concentrations of endogenous molecules, such as nicotinamide adenine dinucleotide (NADH), flavin adenine dinucleotide (FAD), and porphyrins, can be measured using visible light and inherent autofluorescence, requiring no exogenous imaging agent to be administered. Autofluorescence has been investigated for endoscopic cancer diagnosis extensively for the upper aerodigestive pathway in the 1990s and recently has had a resurgence in multispectral imaging for diagnostics and guided surgery.^29^^–^^31^

**Fig. 2.**
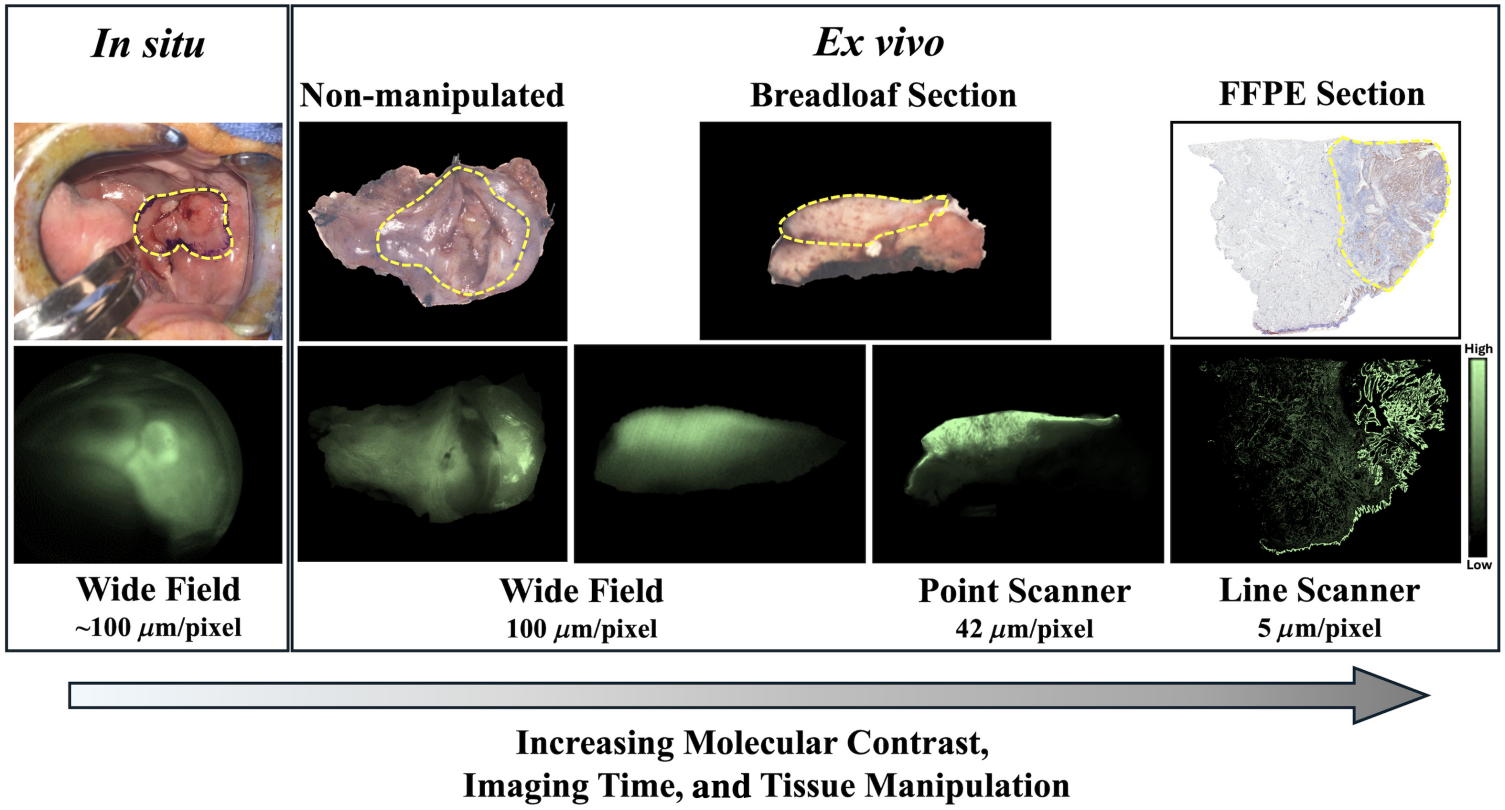
Human head and neck cancer tissues shown in RGB (top) and ABY-029 fluorescence (bottom) were imaged in a number of tissue sample configurations. The tumor location is indicated by a yellow dashed line in the RGB images. *In situ*, wide-field images were collected using a custom-built near-infrared imaging system. Wide-field images of *ex vivo* non-manipulated and breadloaf section tissues were collected with the Perkin Elmer Solaris. Point scanner images of *ex vivo* breadloaf sectioned tissues were collected with the LI-COR Odyssey CLx. Line scanner images of *ex vivo* FFPE sectioned tissue were collected with the LI-COR Odyssey M.

**Fig. 3:**
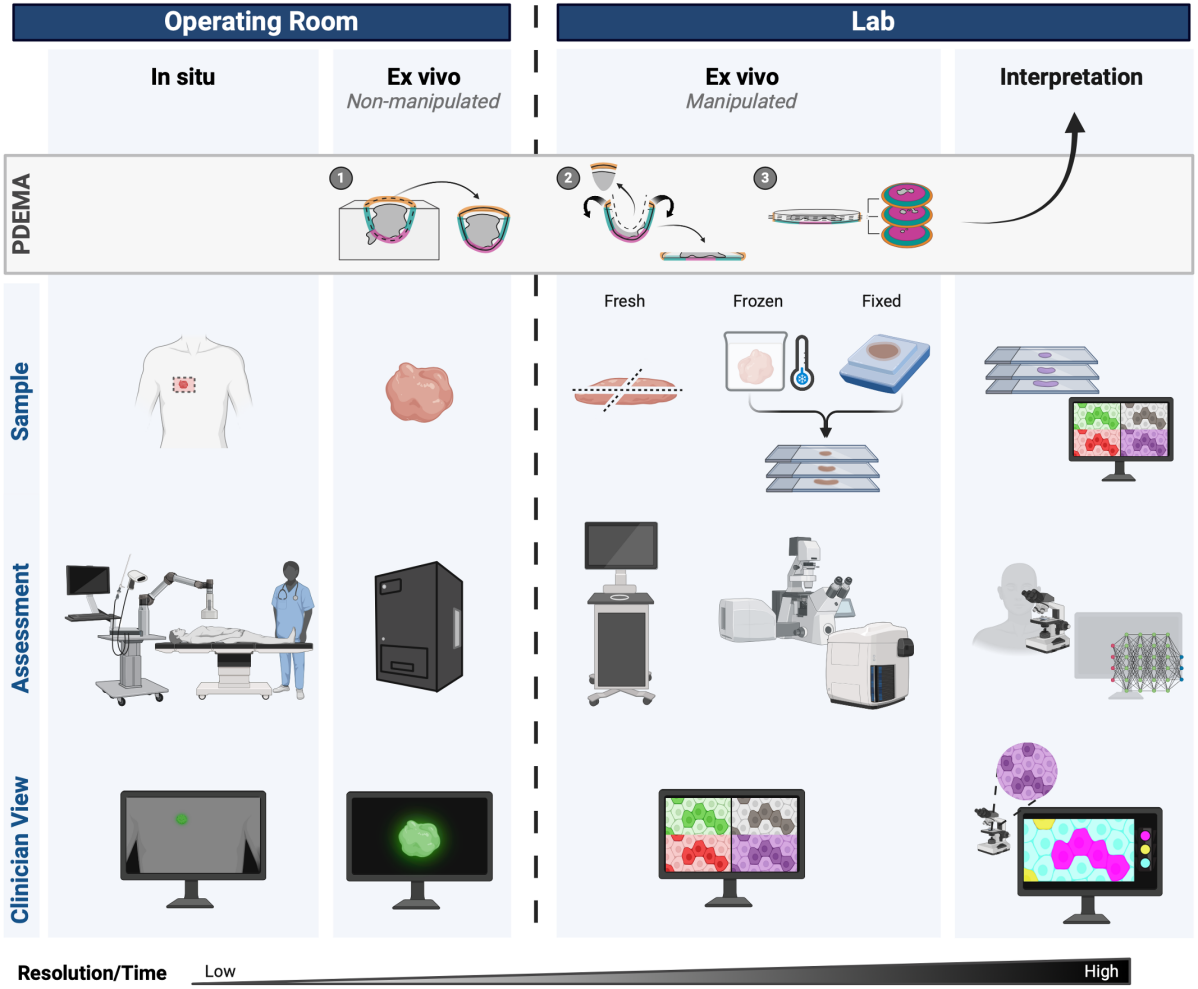
Surgeon (operating room) to pathologist (lab) workflow can incorporate fluorescence molecular imaging at every stage. Standard PDEMA (peripheral and deep *en face* margin assessment) already follows this workflow but without intermediate imaging. Created with BioRender.

Margin assessment of solid tumors using FMI provides advantages over other imaging technologies for the surgery-to-pathology workflow. Fluorescence, whether endogenous or exogenous, is visible in the macro-(patient and whole specimen), meso-(patient and whole or manipulated specimen), and microscale (cell/molecular target), as shown in Fig. 2. As such, FMI can be used to guide tumor detection at micrometer to millimeter depths *in situ* (of primary tumor and residual tumor within the surgical wound bed) and *ex vivo* non-manipulated surgical specimen within the surgical suite (commonly referred to as “back table” imaging), or as *ex vivo* manipulated specimen in the pathology grossing lab (Fig. 3). Fluorescence imaging provides real-time, or near real-time, feedback for surgical visualization and necessary imaging equipment is relatively cost-effective with simple hardware and software as compared with techniques with cumbersome equipment such as MRI or CT. A variety of instrumentation, including wide-field surgical, high-resolution scanning, and microscopy systems, provides imaging capabilities on the centimeter to micron imaging scale. However, one large limiting factor for the clinical implementation of FGS is that accurate interpretation by the surgeon and/or pathologist requires a steep learning curve with high possibilities of misclassifying tissues.

## 3 Margin Assessment Platform (MAP)—A Novel FMI Workflow for PDEMA

To extend the use criteria of PDEMA beyond small, high-risk NMSCs, we propose an imaging platform that combines macroscopic to microscopic imaging along with image interpretation. A margin assessment platform (MAP) that consists of multiple imaging steps that increase both resolution and diagnostic accuracy to rapidly screen tissues may substantially reduce the time for PDEMA, thereby making it feasible for use in solid tumors (Fig. 9). The crux of MAP is that the entire resected tissue does not require real-time histopathological evaluation (the limiting step of PDEMA). Rather, at each imaging step, tissues that are determined to be a true positive result in immediate surgical removal of additional tissue layers, whereas true negative tissues are sent for permanent sectioning—both instances where the tissue is eliminated from the standard PDEMA workflow. The equivocal tissues progress through the MAP pipeline with increasing levels of imaging resolution and diagnostic accuracy for diagnosis.

There are several criteria that a MAP should meet to be successfully incorporated into the PDEMA workflow. First, the FMI technique should be quantitative allowing for the assessment of disease burden in a highly reliable manner across all patients and reducing errors due to physiological differences in tumors. Second, if possible, a single FMI technique is utilized for macro- to micro-scale imaging to reduce the hardware and software required in addition to the education of the staff performing the surgery. Third, the FMI method should be fast to reduce the margin assessment time compared with standard PDEMA. Finally, the FMI technique should clearly output diagnostic values such that the surgeon and surgical team do not have to interpret fluorescence emission intensities, which can often be misleading due to biological variances between patients and tumors. We will discuss a number of clinical and pre-clinical FMI techniques used for fluorescence-guided surgery in the surgical suite and in the pathology lab and will assess their ability to successfully perform PDEMA according to these criteria.

## 4 FMI in the Surgical Suite (*In Situ* and *Ex Vivo* Whole Surgical Specimen)

In standard practice, surgeons rely on preoperative imaging, palpation, and visualization to determine the resection margins during the operation. Traditional PDEMA focuses on the pathological assessment of resected specimens acquired by surgeons, and any *in situ* information relies on the surgeon’s ability to map that information back to the surgical site. On the other hand, FMI enables imaging of both the primary specimen *in situ*, which can guide the surgeon in the location of the tumor and appropriate margin to resect as well as the *in situ* surgical resection bed or the *ex vivo* whole (non-manipulated) excised specimen within the surgical suite that can inform the surgeon of potential positive or close margins resulting in immediate removal of additional tissue. Intraoperative visualization of tumors and residual cancers is guided by a number of imaging systems including wide-field overhead or handheld, endoscopic, *in situ*, and *ex vivo* before processing the excised specimen for further analysis. The ability to use FMI to image the tumor *in situ* and *ex vivo* would be a valuable addition to the PDEMA workflow, as it can improve the accuracy of analysis, aid in reconstructing the tumor back to the patient for subsequent tissue samples, and reduce assessment time.

### 4.1 In Situ

There is a large body of work demonstrating the use of intensity-based imaging of molecularly targeted agents to detect tumors *in situ* in preclinical and clinical settings using a wide variety of targeting agents. For instance, one of the most well-known molecular imaging agents is Cytalux (pafolacianine, OTL38), a folate-derivative that binds to both folate receptor -α and -β with nearly equal affinity and is found overexpressed in a number of cancers. Cytalux was the first receptor-targeted fluorescent molecular-targeting agent approved in 2021 for ovarian cancer cytoreductive procedures^32^ and in 2022 for lung cancer resection. ^33^ The aim of *in situ* Cytalux imaging for both ovarian and lung cancers is the visualization and identification of additional lesions. In ovarian cancer debulking procedures using the EleVision IR (Medtronic, Minneapolis, MN) or Quest Spectrum (Olympus, Tokyo, Japan) imaging system, Cytalux helped surgeons identify additional lesions in 33% of patients with an 83% sensitivity of detection and 24.8% false-positive rate.^34^ In lung cancer resection surgeries using the VS3 Iridium system (part of the EleVision IR Platform), 19% of patients had the primary lung lesion localized via Cytalux fluorescence but not conventional methods. Furthermore, 8% of patients had at least one additional malignant lesion identified over white light imaging. However, it is important to note that margin assessment for lung cancer was not performed *in situ* but using *ex vivo* imaging techniques. Many similar studies have been published in both the pre-clinical and clinical settings, which use FMI to identify and distinguish tumors from normal tissues. This work is highlighted in the aforementioned review articles of FMI for surgical guidance and will not be discussed further here. The work presented within this section specifically looks at margin detection and analysis, with correlative pathological data and not simply identification of tissue types within a field of view.

#### 4.1.1 Intensity-based imaging

The most common fluorescence intensity-based metrics used in the field are mean fluorescence intensity (MFI), median fluorescence intensity, and signal-to-background ratio (SBR) or tumor-to-background ratio (TBR). SBR/TBR is calculated by dividing the average signal in the region-of-interest (ROI), or tumor region, by the average signal found in the surrounding area, or normal region. A threshold is set to differentiate between tumor and healthy regions upon obtaining the metric. Nevertheless, signal quantification methods and establishing thresholds lack standardization, whereas an appropriate threshold is paramount.

**Fig. 4:**
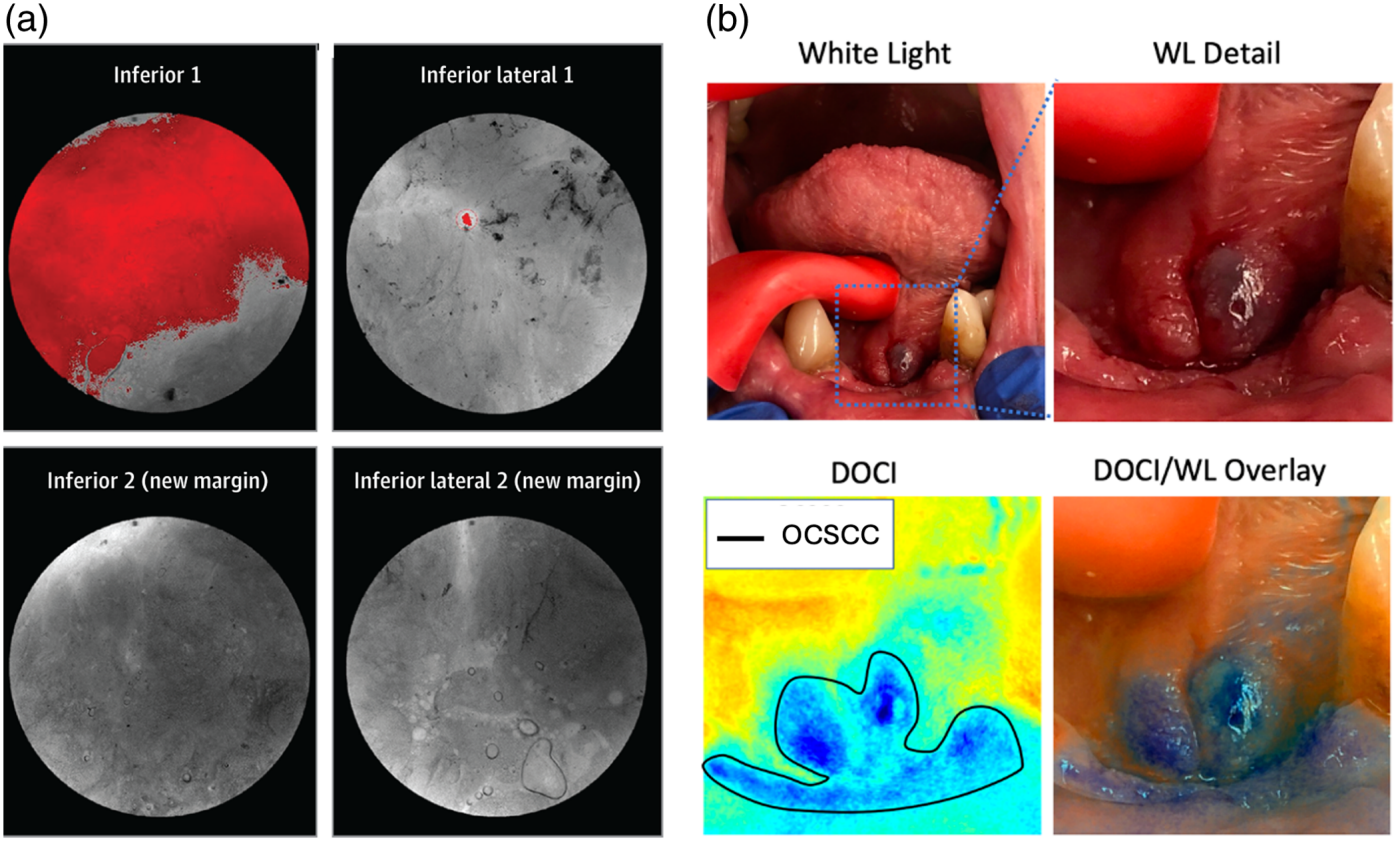
Clinical examples of *in situ* imaging. (a) Intraoperative imaging of the lumpectomy cavity using the Lumicell Direct Visualization System.^35^ LUM015 fluorescence signal, shown in red, was suggestive of residual tumor (top row), and so an additional shave was taken leaving the new margin negative (bottom row).^35^ Adapted from Ref. 35; © Jama Network. (b) *In vivo* dynamic optical contrast imaging (DOCI) of an oral cavity squamous cell carcinoma (OCSCC). DOCI is a label-free tool that makes use of relative fluorescence lifetime values to distinguish tumors from normal tissue.^40^ WL: white light. Adapted from Ref. 40, with permission from John Wiley & Sons, Inc., © 2023 American Academy of Otolaryngology–Head and Neck Surgery Foundation.

In April of 2024, LUMISIGHT™ (pegulicianine, LUM015), a cathepsin activatable fluorescent imaging agent, was approved for imaging cancerous tissues in the resection cavity of breast cancer patients following lumpectomy surgery using the Lumicell™ Direct Visualization System (DVS); together, LUMISIGHT and Lumicell DVS are known as the LumiSystem™ (Lumicell, Newton, MA, United States). Fluorescence detected within the lumpectomy cavity was indicative of residual cancer and thus warranted an additional shave from that region, similar to the process of PDEMA [Fig. 4(a)].^35^ Unlike PDEMA, the observation of residual cancer is being made in the resection cavity, not the surgical margin of the excised specimen. In the phase III multi-center, two-arm, randomized, blinded study (NCT03686215), up to two extra layers of tissue were taken for histopathological analysis. At least one extra layer was taken in 46% of the patients and 7.6% of the total patients had pathologist-confirmed residual cancer. It was shown that 16.1% of participants had positive margins after standard of care and that 14.5% of participants with positive margins after standard-of-care breast-conserving surgery converted to negative after using the LumiSystem. Scanning the entire lumpectomy surgical resection bed and analyzing tissue was shown to add less than 7 min to the whole procedure and resulted in a per-margin analysis^36^ with 85.2% specificity and 49.3% sensitivity.^37^

#### 4.1.2 Fluorescence lifetime imaging

Fluorescence lifetime imaging is a label-free tool that measures the time a fluorophore stays in its excited state before returning to the ground state and emitting a photon. Autofluorescence FLIM makes use of endogenous fluorophores to gain insight into a tissue’s molecular environment because each molecule has a characteristic lifetime that is affected by factors such as pH, ion concentration, lipophilicity, and morphology. Thus, FLIM can be used to detect metabolic and biochemical changes to differentiate between normal and diseased tissue. FLIM has shown particular utility for margin assessment in head and neck,^38^^,^^39^ oral and oropharyngeal cancers.^40^^–^^43^ Duran-Sierra et al. used widefield multispectral autofluorescence FLIM endoscopy to identify 13 features (e.g., intensity, decay rate, metabolic redox-ratio) that could distinguish between dysplastic/cancerous tissue and normal oral epithelial tissue *in situ*.^42^ Then, in a following study on 23 oral cancer patients, a machine learning algorithm was implemented where an AUC of 0.81 was achieved.^41^ Recent work by Tam et al. showed that dynamic optical contrast imaging (DOCI)—a technique that normalizes nine channels of fluorescence decay intensity by corresponding steady-state fluorescence intensity—could successfully discriminate tumors from healthy adjacent tissue intraoperatively [Fig. 4(b)] with minimal imaging time (10 s per channel).^40^ DOCI values (relative fluorescence lifetimes) of the normal tissue all had significantly different values from that of oral squamous cell carcinoma, and resultant images were consistent with corresponding histology but there were no diagnostic values provided.

### 4.2 Ex Vivo, Non-Manipulated

Often called “back table imaging,” the process of imaging the surgically resected specimen prior to any manipulation provides several advantages over *in situ* imaging. The first is the reduction in the volume of the background tissue, which typically has a lower level of fluorophore than the tumor itself, reducing the total pathlength and scattering of photons in the illuminated tissue. This results in an increase in the observable contrast, making the tumor more easily detectable. In addition, the tissue can be imaged in a black box imaging system, which further reduces stray light from the surgical lamps and further increases the observable contrast. However, a major limitation to *ex vivo* imaging of a three-dimensional object is a non-uniform illumination surface that can result in varying intensity based on distance from the camera.

#### 4.2.1 Intensity-based imaging

**Fig. 5:**
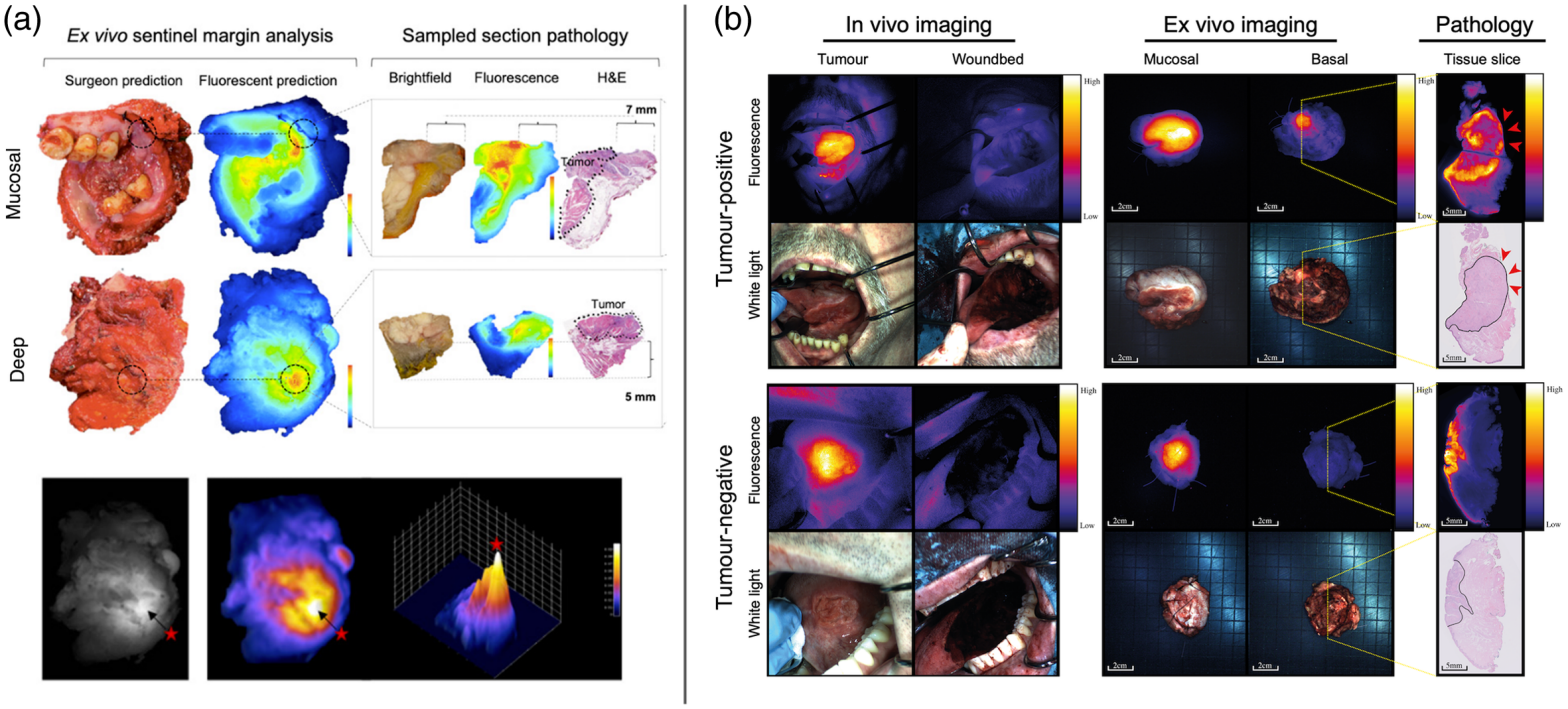
Examples of *ex vivo*, non-manipulated fluorescence molecular imaging for margin assessment. (a) Example of *ex vivo* sentinel margin analysis in an oral squamous cell carcinoma (OSCC) using panitumumab-IRDye800CW.^45^ The sentinel margin was predicted by both the surgeon and fluorescence on the mucosal and deep surfaces of the resected specimen.^45^ Margin distance was confirmed through sectioning of the suspicious regions and comparing fluorescence with hematoxylin and eosin (H&E) staining.^45^ The bottom row demonstrates how maximum fluorescence signal intensity (arrow and red star) was isolated to identify the sentinel margin on the deep surface.^45^ Adapted from Ref. 45, © 2022 Society of Nuclear Medicine and Molecular Imaging. (b) Representative OSCC cases of tumor-positive (top panel) and tumor-negative (bottom panel) margins.^46^ *In vivo* imaging was performed; however, tumor-positive/negative classifications were made based on *ex vivo* imaging.^46^ The strong cetuximab-800CW fluorescence signal was found within the margin of the tumor-positive case, whereas the minimal signal was seen in the margin of the tumor-negative case, which corresponded well with the histopathology.^46^ Red arrows indicate a positive margin, and the solid black line outlines the tumor border. Adapted from Ref. 46, © 2023 de Wit et al.

A method of *ex vivo* margin analysis being adopted in a number of FMI-guided surgery studies is the “sentinel margin” method, first proposed by van Keulen et al. in 2019, using panitumumab-IRDye800CW for head and neck cancer (NCT02415881).^44^ This technique involved imaging the deep surface of the resected specimen, coined “sentinel margin,” with the Pearl Trilogy (LI-COR Biosciences, Lincoln, NE), a closed-field imaging system. The rationale lay in the correlation between the tumor’s proximity to the surgical margin and the corresponding increase in fluorescence signal. In 100% of the cases, the highest intensity peak (utilizing the MFI metric) corresponded to the closest margin to the tumor. This approach took 2.5 min to locate the closest margin on the specimen’s deep surface. A subsequent study by the same group assessed this method using a closed-field, 3D, near-infrared optical imaging system (ELVIS; LI-COR Biosciences, Lincoln, NE) [Fig. 5(a)].^45^ It was shown that a higher diagnostic accuracy was obtained when utilizing MFI to identify the actual sentinel margin (96.4%; p<0.001) than based on surgeons’ evaluation (82.1%; p<0.001). The results indicated a stronger correlation between fluorescence-guided assessment and final pathology than between surgeons’ evaluation and pathological results.

Furthermore, de Wit et al. reported a single-arm, single-center phase II trial (NCT03134846) testing cetuximab-800CW in specimen-driven margin assessment for oral cancer patients using SBR for thresholding [Fig. 5(b)].^46^ Although Explorer Air (SurgVision GmbH, Munich, Germany) was utilized to image *in situ*, no quantifiable margin determinations were made. Margin determinations were made *ex vivo* on the deep surgical margin using two closed-field imaging systems, Explorer Air coupled to a closed-field imaging box and the Pearl Trilogy. Any location where fluorescence emission was observed over the background signal was biopsied. The results showed that an SBR≥2 identified a positive margin with 100% sensitivity and 85.9% specificity. In comparison, an SBR greater or equal to 1.5 could identify close margins with 70.3% sensitivity and 76.1% specificity.

Kennedy et al. reported a three-dimensional near-infrared specimen mapping (3D-NSM) to improve margin assessment during pulmonary ground glass opacities surgery.^47^ Sublobar resections occurred under OTL38 (Cytalux) guidance and *ex vivo* specimens were imaged using the ELVIS. The margin distance was quantified by measuring the distance from the edge of the fluorescent signal from the lesion of interest to the resection margin. Results showed that 3D-NSM identified all tumor-positive and close margins (n=4) while providing a nearly identical report on margin distance compared with what was reported by the pathologist ($R^{2}=0.9362$). 3D-NSM was shown to detect tumor-positive or close margins while reporting the resection margin distance, enabling a rapid check for adequate margin. In the same year, Sarkaria et al. reported that 32 cases with positive margins of 10 mm or less were identified under near-infrared fluorescence imaging using Cytalux, assessed in the operating room by the investigator, and confirmed through histopathology.^48^ Interestingly, in 2023, Azari et al. published a study about OTL38-guided surgery which showed FMI was helpful in guiding surgeons to tumors and assessing margins, but *in situ* TBR measurements did not hold the potential to provide clinically useful information in terms of the nature of cancer as they could not help differentiate between histopathologic subtypes.^49^

In 2020, Lu et al. reported a phase I trial testing panitumumab-IRDye800CW (NCT03384238)^50^ for pancreatic cancer. Although *in situ* imaging was performed using the IRDye800CW-tailored SPY-PHI (Stryker, Kalamazoo, Michigan) imaging platform, the Explorer Air for open-field imaging, and the PINPOINT imaging platform (Stryker, Kalamazoo, Michigan) for laparoscopic imaging, only dose determination measurements were made without pathological confirmation of margin assessment. Fluorescence was shown to discern tumors from normal regions (*ex vivo*) with a sensitivity of 90.3% and a specificity of 74.5%.

### 4.3 Potential for MAP

It is evident that macroscopic FMI in patients *in situ* and large excised specimens *ex vivo* is time efficient, typically only requiring seconds for images to be captured. Intensity-based imaging remains non-quantitative and can be substantially affected by patient and tumor physiology (i.e., vessel perfusion and density, lymphatic drainage). Although sensitivity is often high, specificity is low, indicating that macroscopic FMI will have a high number of false positives. This is not ideal, as minimization of tissue removal is the goal. However, equipment for intensity-based imaging is already incorporated into many operating rooms through commercialized surgical ICG systems, and off-the-shelf black box systems for bench-top imaging are relatively low cost. Multispectral FLIM on the other hand is quantitative and therefore more suitable for diagnostic imaging. The clinical utility of FLIM at the *in situ* and *ex vivo* level remains uncertain as interpretation of AUC of the ROC suggests ≥0.80 as clinically relevant^51^ and current evidence is right at this level (AUC = 0.81). Furthermore, the equipment is not substantially more complicated than intensity-based imaging, requiring a time-gated camera and additional mirrors and filters for separating the wavelengths of light. The current drawback to multi-spectral FLIM is that there are no off-the-shelf systems available, although this may change in the future.

Overall, macroscopic FMI lacks the image resolution required to distinguish small amounts of cancer <1  mm in diameter especially at depth. This is demonstrated in de Wit et al. (2023) where positive margins (0 to 1 mm from the surgical margin) had substantially higher sensitivity and specificity than close margins (1 to 5 mm), where sensitivity was more greatly affected by depth. Even though the normal tissue fluorescence signal may be low compared with the tumor, large differences in volume between the tumor and normal tissue can cause the fluorescence signal contrast to be low. This is due to the volumetric nature of wide-field fluorescence imaging, where diffuse light scatter through the tissue enables the collection of fluorescence from large volumes. This is especially true of near-infrared fluorophores that are often used for surgical guidance. The volumetric effects impede the ability to visualize small regions of cancerous tissue within large background tissue, even when the molecular specificity of the targeted agent is high. The reported diagnostic accuracies are moderate (81% and 82.1%) with moderate-to-high sensitivity (identification of true positive tissues, range 70.3% to 100%) and low-to-moderate specificity (identification of true negative tissues range 49.3% to 85.9%). Thus, these macroscopic methods might substantially misclassify tumor tissue as normal.

Recognizing both the advantages and disadvantages of macroscopic techniques, we propose that some MAP methods may incorporate macroscopic imaging as an initial screening step. Although MAP may provide rapid analysis of large tissue surface areas ($∼75\;{\mathrm{cm}}^{2}$ for a 3-cm long tumor) at several millimeters’ depth, the diagnostic accuracy at this stage of tissue processing and image analysis is low. As such, if macroscopic whole-tissue imaging is proposed, the goal of this high-level screening would be to filter out definitively negative tissue to reduce the number of samples undergoing time-intensive and probably unnecessary pathological evaluation. For our proposed method of MAP discussed in Sec. 7, we will not utilize an *in situ* or *ex vivo* whole, unmanipulated specimen.

## 5 FMI in the Surgical Suite/Lab (*Ex Vivo* Manipulated Surgical Specimen)

Whole, non-manipulated surgical specimen presents some limitations in terms of fluorescence imaging. Varying tissue thickness and uneven surface contours can present challenges in the interpretation of fluorescence images due to collection efficiencies and light scattering and reflectance. To reduce these effects, the *ex vivo* tissue is often manipulated to optimize imaging conditions. For instance, flattening the tissue under pressurized glass and slicing the tissue into smaller sections are examples of tissue manipulation. Moreover, postoperative formalin fixation and tissue sectioning are the most common practices in the pathology lab for processing *ex vivo* tissues for margin assessment. Intraoperative frozen sectioning is also practiced in the pathology lab for more timely margin assessment and can be utilized to improve both the ability to image at higher resolution and reduce errors in imaging due to tissue heterogeneities. Thus, technologies discussed in this section have the ability to bring the meso- to microscopic imaging to the forefront, aiding in the integration of PDEMA into surgery.

### 5.1 Fresh, Whole Compressed

A major limitation of PDEMA is the lengthy tissue processing time (freezing/fixation, sectioning, staining). Optical sectioning via *ex vivo* microscopy offers a promising alternative to achieve the same microscopic resolution but in a fraction of the time because the need for physical sectioning is eliminated. Methods include open-top light-sheet microscopy^52^^,^^53^ MUSE,^54^^,^^55^ two-photon microscopy,^56^^,^^57^ multiphoton microscopy^58^, and autofluorescence microscopy combined with Raman spectroscopy;^59^ however, confocal microscopy (CM) will be discussed in detail here because significant work has been performed clinically. Currently, there are two commercial systems that have been used in the clinic—the Histolog Scanner (SamanTree Medical SA, Lausanne, Switzerland) and the VivaScope 2500M-G4 (Caliber Imaging and Diagnostics Inc., Rochester, New York; MAVIG GmbH, Munich, Germany). Both systems employ fluorescence confocal microscopy (FCM); however, the VivaScope also includes reflectance mode.^60^ In general, CM creates optical sections from fresh, thick-tissue samples using a pinhole to reject out-of-focus light. The procedure still requires some tissue preparation before imaging: grossing to accommodate the system’s field-of-view, staining (e.g., acridine orange, toluidine blue, acetic acid), and sample mounting and flattening. To aid with interpretability, each system also provides pseudocoloring of the display image to mimic standard H&E. The Histolog Scanner (HS) makes use of a purple color scheme, whereas the VivaScope combines reflectance signal with the fluorescence to include more structural information and cellular detail, to be able to generate a two-toned (pink and purple) image that more closely resembles H&E stains.

**Fig. 6:**
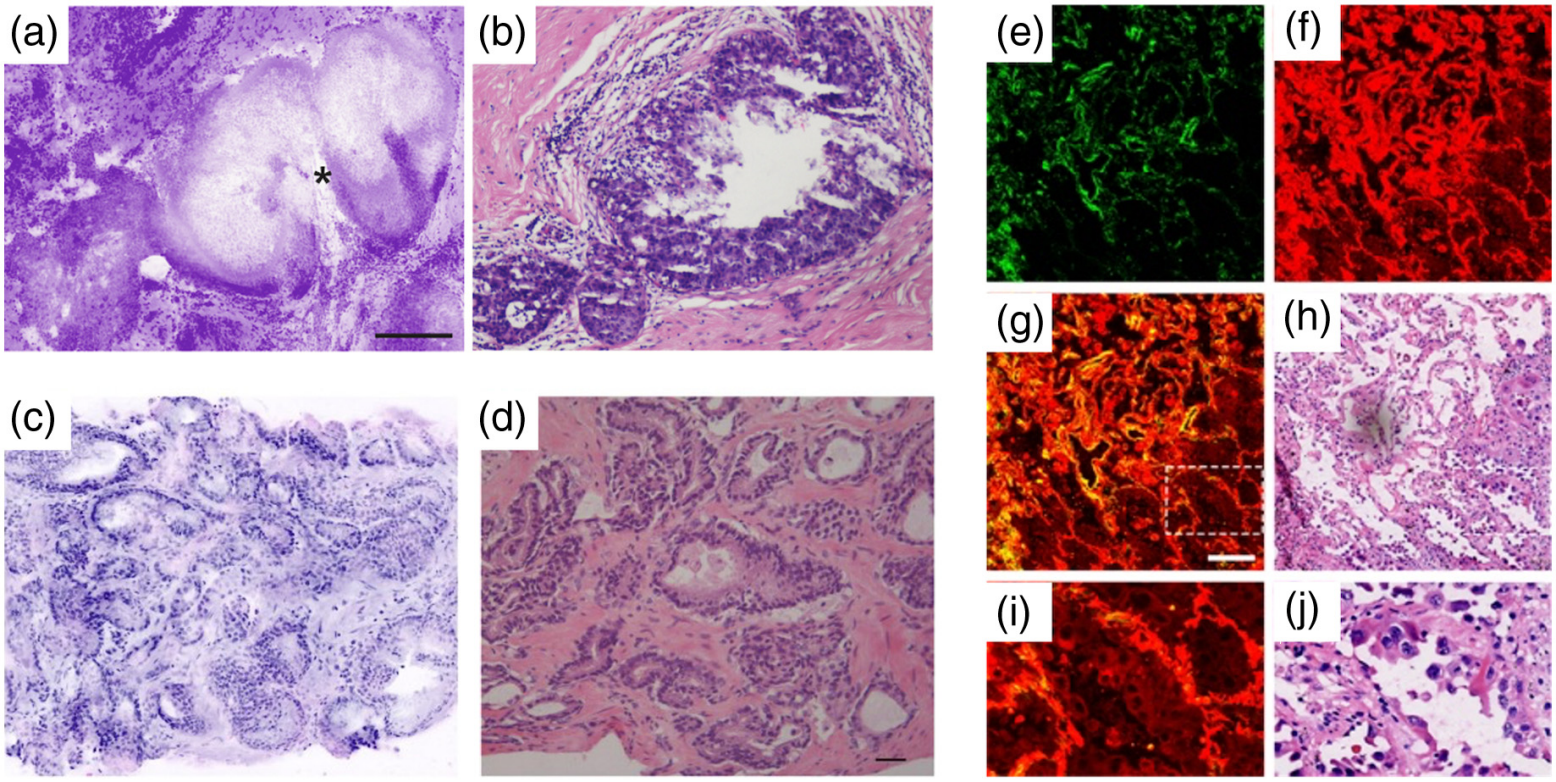
Examples of *ex vivo*, manipulated imaging for margin assessment. (a) False-colored purple fluorescence confocal microscopy image of freshly excised ductal carcinoma *in situ* (indicated by *) scanned using the Histolog Scanner, with the corresponding (b) hematoxylin and eosin (H&E) stain.^61^ Scale bar=250  μm. Adapted from Ref. 61, © 2022 M. Sandor et al. (c) Fresh *ex vivo*, grade 2 prostatic acinar adenocarcinoma scan using the VivaScope 2500M-G4.^65^ Fluorescence and reflectance confocal microscopy are combined to generate a two-toned pseudo-colored image to better mimic H&E. (d) Confirming pathology of panel (c). Scale bar=100  μm. Adapted from Ref. 65, © 2020 Springer-Verlag GmbH. (e)–(j) Frozen section multiphoton microscopy imaging of lung adenocarcinoma. (e) False-colored second harmonic generation (SHG) image, (f) false-colored two-photon excitation fluorescence (TPEF) image, (g) overlaid SHG and TPEF image, and (h) corresponding H&E.^76^ Panels (i) and (j) are magnified views of the dashed white box in panels (g) and (h), respectively. Scale bar=100  μm. Adapted from Ref. 76, with permission from John Wiley & Sons, Inc., © 2023 Wiley-VCH GmbH.

With its relatively large field-of-view (8×8  cm), the Histolog Scanner has found good utility for imaging lumpectomies in breast-conserving surgeries [Figs. 6(a) and 6(b)].^61^^–^^64^ In a recent study by Wernly et al. (2024), it was demonstrated that unnecessary recuts could be reduced from 29% to 8% if margin assessment was informed by HS images versus the standard of care. Moreover, these results were achieved in significantly less time (standard radiography: ∼22  min, HS: ∼13.5  min).^64^ Work by Conversano et al. (2023) also reported time-savings with tissue processing and imaging times of 8 to 10 min (∼4−5  min per 20 cm^2^ section).^63^ The overall findings for several single-center studies showed comparable levels of sensitivity and specificity with the standard of care (radiography plus clinical impression) when surgeons and pathologists interpreted HS-acquired images. On average, sensitivity tended to be lower than the clinical routine, whereas specificity was slightly higher; and pathologists generally outperformed the surgeons. Interestingly, diagnostic accuracy, particularly for surgeons could be improved after a short training period. A common critique of the “virtual stains” produced from optical sectioning is the associated learning curve needed to read the images. Researchers and clinicians from multiple centers across Europe sought to address this with the HIBISCUSS project (high-resolution imaging for breast carcinoma detection in *ex vivo* specimens after breast-conserving surgery by histolog scanner).^63^ In this study, an online training program was developed to prime readers in identifying cancerous versus normal breast tissue in FCM images. With 152 min of average total training time per physician (distributed over nine training sessions), the surgeons’ accuracy and sensitivity significantly increased from 83% to 98% and 81% to 97%, respectively, over seven rounds of evaluations. This brought their performance closer to that of the pathologists, which was relatively consistent over all rounds (average 99.6% accuracy, 99.9% sensitivity).

Similarly, work by Bertoni et al. (2020) revealed a short learning curve for assessing FCM images of radical prostatectomy biopsy specimens collected with the VivaScope [(Figs. 6(c) and 6(d)].^65^ Two FCM evaluations were performed 90 days apart, and diagnostic performance improved substantially between the two—increased accuracy from 89% to 95% and improved area under the receiver operating characteristic (ROC) curve (AUC) of 0.87 to 0.93. An initial investigation comparing prostatic biopsy FCM and matched histopathology showed a positive correlation (Pearson coefficient 0.536, p<0.001) in the pathologists’ confidence between FCM and gold standard diagnosis.^66^ This supports the ability of FCM to mimic H&E staining. Rocco et al. (2021) built off these studies to directly evaluate surgical margins in robot-assisted radical prostatectomy using the VivaScope.^67^ The goal was to present an alternative to NeuroSAFE (neurovascular structure-adjacent frozen-section examination), an intraoperative frozen-section analysis technique proven to increase nerve-sparing and decrease positive surgical margin rates. Similar to the systematic approach of PDEMA, NeuroSAFE is limited by lengthy processing times and the need for dedicated facilities and expert personnel. The work by Rocco et al. showed FCM to be a promising solution with all patients having negative surgical margins (even after secondary resection) after final histopathology. Furthermore, after 1-year follow-up, nine out of ten patients were disease-free, nine were fully continent, and four out of five recovered sexual function. It is interesting to note that the authors also utilized and recommended the use of *en face* or “Mohs sections” (as referred to in the work) instead of the breadloaf sections typically used in NeuroSAFE. This may have contributed to the positive outcomes and underscores the utility of *en face* sections for comprehensive margin analysis.

As one of the first adopters of CM, there have been numerous demonstrations of successful rapid margin evaluation of skin cancers.^68^ Basal cell carcinomas (BCCs), in particular, have been extensively investigated with reports of 88–96.6% sensitivity and 89.2–99% specificity.^60^ In 2014, Bennàssar et al. presented the first prospective study where FCM was implemented daily for Mohs surgery of BCC and assessed the time impact.^21^ This study boasted 88% sensitivity, 99% specificity, 98% positive predictive value (PPV), and 97% negative predictive value (NPV), all while significantly reducing tissue processing time (FCM: 10.1±1.22  min, standard frozen-section H&E: 28.2±2.2  min).

One limitation of optical sectioning with microscopy is that results are strongly dependent on the quality of tissue preparation. As previously mentioned, samples (whole or grossed) are flattened and/or mounted between glass slides for imaging. It is vital that the tissue makes tight and complete contact with the surface for comprehensive interrogation and to avoid artifacts, e.g., air bubbles and tissue folds; failure to do so increases the likelihood of false negatives.^60^^,^^69^^,^^70^ Standard techniques of flattening include applying pressure to the specimen with surgical tools for the open-air Histolog Scanner,^61^^,^^63^^,^^71^ or securing it with silicon glue or modeling clay between microscopy slides, as recommended for the VivaScope.^65^^–^^67^ Unfortunately, this only applies homogenous compression on samples that have variable tissue composition, density, and thickness. As a result, achieving perfectly flat samples is a challenge and whole tissue CM remains susceptible to false negatives. Some groups have tried to combat this with custom-built mount and flattening devices that make use of a sponge and magnet combination^70^ or an array of grub screws that can be individually adjusted to tailor the amount of compression for different tissue regions.^69^ Although these techniques showed improvement in image quality, they introduce an additional step and rely on user discretion. In addition, whole-tissue optical imaging is depth-limited. Although it can offer profound time-savings, the information obtained is largely surface-weighted. The VivaScope, for instance, has a depth penetration of 200  μm.^60^

### 5.2 Fresh, Cross-Sectioned

As described in the introduction, standard pathological workflow involves sectioning the surgical specimen into cross-sectional breadloaf (Fig. 1) or radial slices to sample the surgical margin. Typically, the surgical specimen is cut into three or more sections, which are millimeters thick. These sections, further reduce the volume of tissue that can be imaged and thus typically increase contrast, although the fluorescence signal itself may decrease. Benefits of sectioned, fresh tissue include the ability to image the entire depth if using wide-field illumination and millimeters thick tissue, as compared with both *in situ* and *ex vivo* whole specimen imaging. Correlation to pathology at this stage is generally more straightforward because pathological tissue sections are created from the same face that is imaged. However, pathological sections represent only ∼4  μm of the entire sample, whereas fluorescence imaging at long wavelengths (i.e., NIR) is capable of imaging tissue that is several millimeters thick in contrast to optical sectioning in the previous section that is only capable of imaging a few hundred microns in depth.

#### 5.2.1 Intensity-based imaging

A number of clinical trials image cross-sectioned breadloaf slices of the fresh surgical specimen prior to formalin fixation to confirm the distance of the tumor from the deep surgical margin. This has been performed to confirm sentinel margins determined from imaging the whole, fresh peripheral, and deep margin as described in Sec. 3.2.1. Fluorescence images of the fresh cross-sectioned tissue (Fig. 5) have been found to match pathologist-determined margin distances.^45^^,^^46^ However, several aspects should be considered when comparing breadloafed tissue slices to histopathological sectioned tissue. The first is that alignment of the fluorescence signal to the pathological sections may be difficult due to dehydration and deformation of the tissue that occurs during fixation. In addition, breadloaf sections are typically several millimeters thick, whereas H&E and IHC stained sections are 4–10-m thick; therefore, fluorescent structures that are visible in the fluorescence images may not be observable in a single pathological section. This can be accounted for by staining step-sectioned tissues and rendering a 3D pathological volume that more closely matches the fluorescence images of the fresh tissue.^72^ However, this process is too cumbersome for the clinical pathology workflow but can be used preclinically to validate fluorescence methodologies.

#### 5.2.2 Paired-agent imaging

Studies have been conducted to test utilizing dual agents in imaging to enhance tumor detection while correcting for the nonspecific and varied signals in FGS. Paired-agent imaging (PAI) involves the coadministration of untargeted and targeted imaging agents while utilizing a new quantitative term, binding potential (BP), that is correlated to the concentration of the target molecule multiplied by the affinity of the receptor-target binding. BP can be calculated kinetically using the reference tissue model or using single time point measurements determined by rationing the fluorescence intensities of the targeted and optically corrected untargeted agent minus one. Notably, BP has been demonstrated to be comparable to *in vivo* immunohistochemistry (IHC) for the targeted protein^73^ and has been shown to detect as few as 200 cells in breast cancer lymph node metastases.^74^ PAI can be performed with the same equipment as intensity-based imaging, as long as there are multiple fluorescence channels. For instance, the Solaris (PerkinElmer, Waltham, MA) and Pearl Impulse have been used in PAI studies. Notably, BP provides a quantitative measure, unlike fluorescence intensity, and so is less susceptible to volumetric and dosing effects. BP may be able to provide a universal metric for a tumor to normal tissue thresholding, as fluorescence intensity alone has not been able to be utilized between patients, imaging systems, and institutions.

Wang et al.^75^ evaluated PAI using ABY-029, a fluorescent anti-EGFR (epidermal growth factor receptor) Affibody molecule, as the targeted agent and IRDye 700DX carboxylate as the untargeted agent in excised and bisected oral tongue squamous cell carcinoma tumors in a xenograft mouse model. The bisected specimen would closely mimic the breadloafed gross tissue specimen in the human workflow. The study, performed on mice implanted with three types of head and neck squamous cell carcinoma, showed a significant increase in diagnostic accuracy compared with a single-agent imaging (0.908 versus 0.822, respectively). The detection power of PAI was especially notable in cancer types that expressed low levels of EGFR and highly heterogeneous regions. In addition, PAI was able to distinguish tumors from normal structures that endogenously expressed EGFR, such as salivary gland, whereas single-agent imaging was not. A later study utilizing ABY-029 and IRDye 680LT performed PAI on *en face* margins cut from the primary surgical specimen commonly used in PDEMA for NMSCs. The *en face* margin reduces the volume of tissue that is required to the frozen section, whereas the debulked primary tumor is sent for permanent sectioning. Torres et al.^72^ assessed the use of fluorescence for identifying tumor-positive margins in whole surgical specimens and the corresponding *en face* margin. Both single-agent imaging and PAI were compared using the Pearl Impulse imaging system. Results indicated that *en face* margin consistently outperformed the whole surgical specimen for both single-agent imaging and PAI, whereas PAI was superior to single-agent imaging regarding diagnostic accuracy. Both mean and max PAI BP facilitated the achievement of an optimized threshold to distinguish tumors from normal tissue, leading to a 100% diagnostic accuracy determined from the ROC-AUC, whereas the single-agent scheme only achieved 97–98% accuracy. Contrast-to-variance-ratio (CVR) was also increased by 4.4±1.5 times for all tumor tissues using BP, and max BP offered the highest average CVR. PAI BP had the strongest concordance with histopathological results when determining the edge of the tumor using line profiles. The use of *en face* margin for imaging residual cancer has two benefits over the sentinel margin method using the whole surgical specimen discussed in Sec. 3.2.1. The first is that the total volume of the surgical specimen is decreased, reducing the total pathlength of photons traversing the specimen, which has been previously demonstrated to increase contrast. The second is that removing the bulk tumor reduces the level of background fluorescence emitted from the sample, thus making small amounts of residual tumor more easily visualized.

### 5.3 Fresh, Frozen Sectioned

Touted for its speed compared with permanent fixation, frozen-section histopathology is routinely used for intraoperative margin analysis of several cancers (head and neck, breast, skin, etc.). The thin slices permit subcellular resolution and information on tissue structure that is lost in the tradeoff of time-reduction with whole slide imaging (WSI). Samples can be snap-frozen and sectioned within minutes (∼15  min) yet staining and slide reading remain the limiting factors. Often used to confirm negative margins in solid tumor resections, as commonly performed in oral head and neck cancers, frozen sectioning plays an integral role in PDEMA of NMSCs. Using frozen sectioning for PDEMA is often limited to smaller tumors in critical regions (i.e., head, neck, genitals) due to the time-consuming nature of sectioning the entire surface area of the peripheral and deep tumor margin. Translation is limited for whole margin assessment for solid tumors due to these time constraints.

#### 5.3.1 Multiphoton microscopy

Multiphoton microscopy (MPM)—composed of two-photon excitation fluorescence (TPEF) and second harmonic generation (SHG)—is a promising tool that can image tissues label-free.^76^^,^^77^ Note that a significant advantage of MPM is its penetration depth of up to several hundred microns, and as such, thick-tissue imaging can be achieved;^58^ however, its use for frozen sections will be discussed here. The nonlinear excitation process permits autofluorescence imaging of structures such as NADH, FAD, and elastin; and extracellular matrix components such as collagen generate strong SHG signal. Xi et al. leveraged this to delineate tumor boundaries in early-stage lung adenocarcinoma.^76^ Overlaid TPEF and SHG images revealed similar cell morphology and tumor microenvironments compared with corresponding H&E [Figs. 6(e)–6(j)] without any need for exogenous agents and within ∼8  min of imaging time per tissue section. A machine learning algorithm was used to generate a “collagen score” to distinguish between normal and cancerous tissue at tumor boundaries, and the classifier had a sensitivity, specificity, PPV, and NPV of 96.7%, 94.2%, 94.6%, and 96.4%, respectively.

#### 5.3.2 Paired-agent imaging

The contrast of exogenously administered FMI agents increases substantially as the volume of tissue decreases and the resolution of imaging increases. Therefore, imaging frozen sections after cutting but prior to pathological staining was investigated by Wang et al. (2022) using the Odyssey M scanning system (LI-COR Biosciences, Lincoln, NE) that allows for pixel resolutions ranging from 5 to 100  μm/pixel.^78^ The Odyssey M can scan entire slides unsupervised (entire 25  mm×75  mm slide) in two fluorescence channels at a rate of 1.2  min/slide at 100  μm/pixel resolution and 11.2  min/slide at 5  μm/pixel resolution. However, considering a supervised scan with a 2.5-min pre-scan and selection of 4 cm^2^ ROIs, the scan time per slide is reduced to 4.2 and 0.9  min/slide for 100  μm/pixel and 5  μm/pixel resolutions, respectively. Both single-agent imaging of ABY-029 and PAI using IRDye 680LT as the untargeted agent demonstrated that PAI outperformed ABY-029 alone for all tumor types. Diagnostic accuracy determined by the ROC-AUC was found to range from 0.79 to 0.91 for PAI of low and high expressing tumors, and 0.6 to 0.82 for ABY-029 of the same tumor lines. Furthermore, the contrast determined by CVR was also higher for PAI (0.6 to 1.1) as compared with ABY-029 alone (0.1 to 0.8).

### 5.4 Formalin-Fixed, Sectioned

Permanent-fixed histopathology is the gold standard for diagnosis. The high resolution and quality, however, come at the cost of excessively long tissue processing times (>24  h). Initial fixation accounts for a large portion, but similar to frozen sections, paraffin-embedded sectioning is also limited by staining and reading times, which are typically >24  h and can even be weeks depending on the workload of the pathology department.

#### 5.4.1 Intensity-based

Formalin-fixed, breadloafed surgical specimens were assessed in the previously discussed phase I trial testing panitumumab-IRDye800CW (Sec. 3.2.1) for pancreatic cancer prior to paraffin embedding and sectioning.^50^ The fluorescence intensity of the formalin-fixed tissues was assessed for a positive margin, defined as 1 mm from the surgical edge. It was found that a 3.5 times reduction in fluorescence intensity was observed within 1 mm from the cut edge in patients with positive surgical margins, whereas the fluorescence signals remained consistent over the same 1 mm distance for patients with negative margins. Although formalin-fixed tissues retain fluorescence from IRDye 800CW,^79^^,^^80^ it has been shown that the fluorescence can decrease substantially over the initial 24 h period also affecting measured TBRs.^80^ However, fluorescence appears to remain stable after 24 h,^79^ and the fixed tissue will more closely match pathology assessment because the shrinking effects of formalin have already occurred. Although demonstrated in fixed tissue, validating the measurement in fresh, grossed tissue sections would considerably increase the speed of margin assessment and could benefit the patient during surgery by removing additional tissue in the positive margin region.

#### 5.4.2 Autofluorescence multispectral imaging

Autofluorescence multispectral imaging in formalin-fixed, paraffin-embedded (FFPE) samples showed that the technique could distinguish between normal tissue, pterygium (a benign ocular surface disorder), and ocular surface squamous neoplasia in optical biopsies.^81^ Multispectral images were collected over 59 channels to capture spectral signatures of endogenous fluorophores such as NADH, FAD, protoporphyrin IX, and elastin in each tissue type, and a machine learning algorithm was used to automatically classify biopsy samples with an accuracy, sensitivity, and specificity of 88%, 84%, and 91%, respectively. The machine learning algorithm used a fused classification framework that combined a previously developed intra- and inter-patient classification framework from quantitative hyperspectral imaging.^82^ The combination of label-free image acquisition (∼47.2  s per image, average 0.8 s per channel) and automated analysis support the potential for real-time diagnosis using autofluorescence multispectral imaging. Although image collection is fast, processing tissue in FFPE sections is time-consuming and generally on the order of days.

### 5.5 Potential for MAP

Meso- to microscopic FMI is typically more time-consuming than macroscopic FMI due to required tissue processing (manipulation of fresh tissue, frozen sectioning, or FFPE) and high-resolution image collection. There are commercially available FCM systems, but the integration into clinical practices is not widespread, likely due to the high cost of the system and the specialized skills required to successfully prepare tissues and interpret results. Therefore, it is unlikely that FCM will become ubiquitous in clinical settings without substantial decreases in cost and automation. Furthermore, although FCM demonstrates superb diagnostic abilities, the depth of imaging is restricted to only a few hundred micrometers. Multi-photon microscopy has similar pros and cons to FCM, with expensive and highly technical equipment, superior detection capabilities but limited depth penetration, which limits its wide-spread usability. Single-agent intensity-based and paired-agent imaging both use easily accessible commercialized systems that are readily available and are often already used in clinical trials across the world. Both wide-field black box and scanning systems are available to span a range of resolutions and wavelength channels with easy user features. Paired-agent imaging would require additional software to convert fluorescence images into quantitative binding potential maps but otherwise would integrate easily into the surgery-to-pathology workflow. This has already been demonstrated by a number of clinical trials using commercialized systems. Comparatively, the cost of intensity-based and pared-agent imaging is low and technical adaptably high.

Molecular contrast increases with increasing resolution^83^ (Fig. 2) and as such, diagnoses with higher sensitivity than macroscopic imaging can be made; however, this comes at the expense of time, especially in large diameter tumors (e.g., the total surface area of the margin would be $∼75\;{\mathrm{cm}}^{2}$ for tumors 3 cm in diameter). We have demonstrated throughout Secs. 4 and 5.1 to 5.4 that low-resolution imaging of *in situ* and fresh *ex vivo* tissues generally have both moderate-to-high specificity and sensitivity. Here, wide-field imaging could be used for rapid screening of pathologically negative tissues, which could reduce the total amount of tissue that moves on to higher resolution. Positive or equivocal tissues that require higher resolution imaging for more precise diagnostic accuracy can then be prioritized. Furthermore, incorporating fresh tissue manipulation methods such as reducing overall tissue volume by isolating the *en face* margin has been demonstrated to increase the contrast of macroscopic *ex vivo* imaging (for both single and paired-agent imaging) and improve diagnostic accuracy.^72^

As a result, our proposed method of MAP would include mesoscopic widefield imaging of the *en face* specimen margin using PAI, as it is both quantitative and has demonstrated high diagnostic accuracy. This will maintain fairly rapid imaging and analysis time while stratifying positive, equivocal, and negative tissues. Therefore, only a fraction of the entire margin (equivocal) will require microscopic imaging in the second step, which is more time-intensive due to the high resolution required. For PAI, high-resolution scanning can be performed on the *en face* margin or on frozen sections, where high diagnostic accuracy and attention to depth will be important. Again, quantitative analysis of microscopic scans can stratify tissues into positive, equivocal, and negative categories. This will further reduce the number of tissue sections that progress to pathological assessment. Utilizing contrast metrics and/or quantitative imaging methodologies in both the first and second step, such as paired-agent imaging, reduces the interpatient variability and provides a standardized scale for determining diagnostic thresholds.

## 6 Interpretation of Fluorescence Molecular Signal for Margin Assessment

Extensive comprehensive reviews of different FGS systems and their performance have been reviewed previously.^84^^,^^85^ Typically, *in situ* fluorescence-based systems are open-air wide-field systems that are held in place via a surgical arm, hand-held systems utilized by the surgical team, or endoscopic/laparoscopic systems that are either associated with a surgical system, or hand-held. The benefit of these systems and *in situ* imaging is the flexibility and mobility of the system for image collection. *Ex vivo* systems are often the same as the *in situ*, or closed, black-box wide-field systems such as the Pearl Impulse (LI-COR Biosciences, Lincoln, Nebraska, United States) that reduce the background level of light being collected from the surgical room.

Surgeon interpretation of fluorescence images is a limiting factor to clinical implementation. Wide-field (open or closed) and endoscopic/laparoscopic systems tend to suffer from collection efficiency of fluorescence emission and blooming or blurring of fluorescent structures due to scatter within the tissue and the large depth of field, especially in the NIR wavelengths. A thorough understanding of off-target binding, volumetric effects, and optical effects such as reflections and tissue optical properties all limit a clear interpretation of observed signals. To integrate FMI into the clinical setting, the learning curve for surgeons and pathologists needs to be minimized, and the percent probability of tumor on a scale of 0% to 100% rather than raw MFI signal or images of contrast must be mapped in a format that is readily available and interpretable. There are a number of studies that have undertaken probability mapping as a way to report tumor mapping and ease decision-making.

**Fig. 7:**
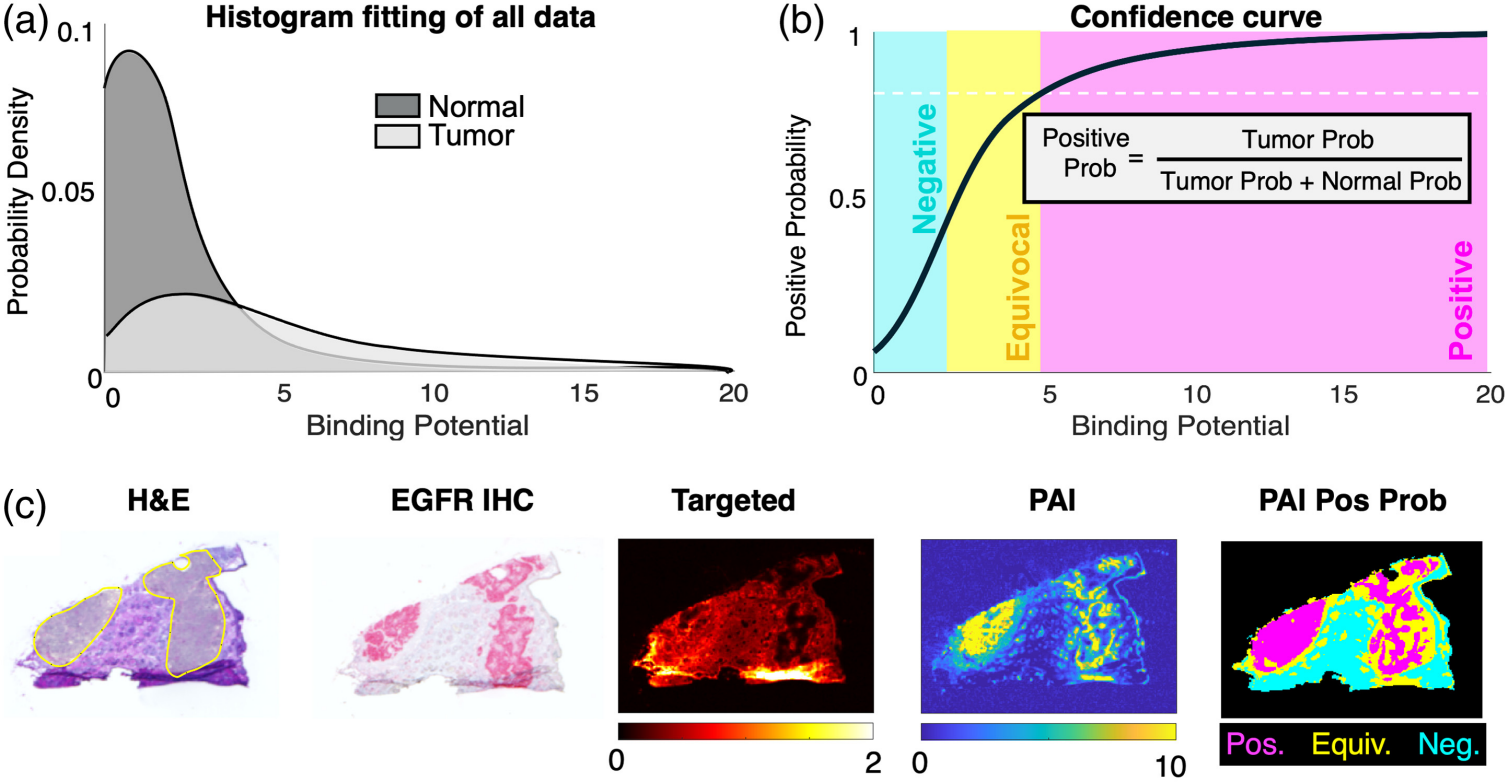
Percent probability confidence mapping. (a) Probability density function of all binding potential pixel intensities used to generate (b) a positive probability confidence curve with positive (≥80%), equivocal (50% to 80%), and negative (≤50%) thresholds. (c) Demonstration of percent probability confidence mapping applied to frozen sections of mouse xenograft squamous cell carcinoma. H&E: hematoxylin and eosin; EGFR IHC: epidermal growth factor receptor immunohistochemistry; PAI: paired-agent imaging.

Our research group has demonstrated the use of percent probability confidence mapping using both pre-clinical (single and paired-agent imaging) and clinical (single agent) fluorescent images. In the pre-clinical setting, we demonstrated a simple method of utilizing consolidated cohort image data to determine and map the positive probability (PP) using the quantitative PAI BP signal and fluorescence signal alone.^78^ Probability histograms were created for both tumor and normal tissue from the entire range of pixel intensities from three xenograft tumors implanted in mice. The PP was subsequently calculated for the entire range of pixel intensities by dividing the tumor probability by the sum of the tumor and muscle probabilities [Fig. 7(a)]. Confidence maps were created by converting the pixel intensities to PP [Fig. 7(b)] and setting a threshold value of 80% PP increased the contrast-to-noise ratio between 20% and 50%. However, it should be noted that conversion of pixel intensity to PP did not alter the AUC of the ROC curve, which was previously discussed in Sec. 4.3.2. Confidence maps of PP for PAI BP in tumors with low EGFR expression were able to visualize tumors with 80% confidence, whereas fluorescence intensity of ABY-029 alone was not.

**Fig. 8:**
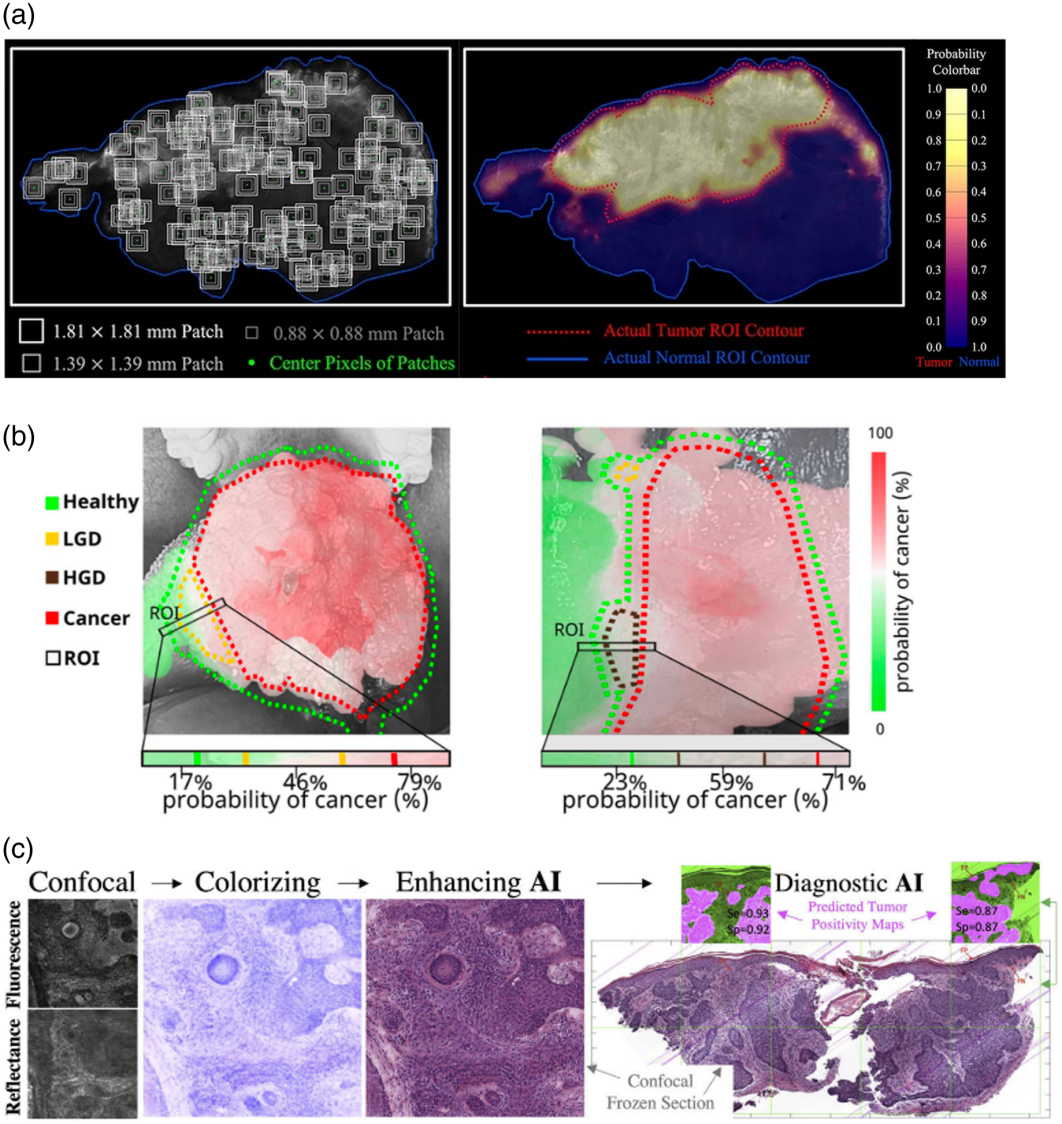
Methods of signal interpretation for clinical decision making in margin assessment. (a) Optomics probability map of a head and neck squamous cell carcinoma imaged with ABY-029.^86^ Three patch sizes (left) were used to generate a continuous tumor probability map (right). Adapted from Ref. 86, © 2023 Chen et al. (b) A fluorescence lifetime imaging-based classifier was used to produce a prediction probability of cancer in intraoperative margin assessment of low-grade dysplasia (LGD, left) and high-grade dysplasia (HGD, right) in head and neck cancer.^38^ The red-to-green probability map is overlaid onto the grayscale image, and color-coded–dotted lines represent the boundaries of healthy, LGD, HGD, and cancer based on annotated histopathology.^38^ ROI: region of interest. Adapted with permission from Ref. 38, © 2023 IEEE. (c) Artificial intelligence was used to enhance the visualization and interpretation of confocal microscopy images of basal cell carcinoma.^88^ Predicted tumor positivity maps were generated to automate diagnosis.^88^ Se: sensitivity; Sp: specificity. Adapted with permission from Ref. 88, © Optical Society of America.

In the clinical setting, we performed a radiomics analysis, termed “optomics,” of 12 patients who received ABY-029 in a phase 0 clinical trial of head and neck cancer.^86^ Fluorescence images of breadloafed primary specimens were collected from 24 specimens on the Odyssey CLx (LI-COR Biosciences, Lincoln, NE) at 42  μm/pixel, divided into a total of 20,073 sub-image patches, and were randomly divided into training and data sets (75%:25%) before all sets were aggregated. The 25 top-ranked features were selected from 1472 standardized radiomic features extracted from each patch and evaluated by minimum redundancy maximum relevance feature selection. It was found that the optomics analysis improved the prediction accuracy and false positive rate (FPR) on all testing slices compared with fluorescence intensity thresholding based on the optimum cutoff point (OCP) of the ROC curve. The accuracy of detection was increased from 0.81 to 0.89 from fluorescence intensity thresholding to optomics, respectively, and the FPR was decreased from 0.21 to 0.12. Confidence maps were produced from the probability of tumor determined from the average of three patch size sampling [Fig. 8(a)], which demonstrated reduced tumor probability in normal regions, as compared with confidence maps produced from single patch sizes.

Another example of mapping probability of tumor arises from autofluorescence FLIM measurements taken from the oral cavity in 85 patients over the period of 2016–2021 by the Marcu research group. Development of a machine learning model to account for the diverse anatomical and biochemical composition of tumors of the oral cavity and oropharynx has been developed over a number of stages.^38^^,^^43^^,^^87^ Briefly, the classifier was trained based on binary classification from histologically confirmed FLIM data of “tumor” and “cancer.” The team developed a binary probabilistic classification model to predict the probability that each measured FLIM point was cancer and then was further classified into anatomy-specific features using a handcrafted feature-based classification model (decision tree, support vector machine, multi-layer perception model, and bootstrap aggregation of the best-performing model to generate ensemble learning model) and non-handcrafted feature-based classification model (convolutional neural network and optimal transport). This allowed confidence maps of “healthy vs tumor” and “dysplasia tested” to be created [Fig. 8(b)].

Optical sections, similar to those produced from confocal microscopy (Sec. 4.1), aim to mimic H&E stains. As such, artificial intelligence (AI) can be leveraged in two ways: (1) improve the visualization of optical sections such that they more closely resemble H&E and pathologist relearning is reduced or (2) eliminate human interpretation altogether and provide diagnostic mapping, such as the techniques mentioned above. Combalia et al. (2021) pursued both approaches by generating AI-enhanced CM images, as well as automated diagnosis, which achieved 88% and 91% sensitivity and specificity, respectively [Fig. 8(c)].^88^ Image enhancement was achieved through style transfer with a cycle-consistent generative adversarial network (CycleGAN) that was trained on 759 patches from various confocal microscopy slides and 282 patches from an H&E slide of human BCC. Binary predictive tumor maps were generated using a U-Net, deep neural network image segmentation model that distinguished BCCs from normal tissue.

### 6.1 Incorporating into PDEMA

The expertise required for the interpretation of FMI signals on both a macro- and microscopic scale is high and not universally taught to surgeons during training. The integration of machine learning, artificial intelligence, and other interpretive image readouts is essential for seamless clinical integration. Therefore, it is likely that a combination of imaging methodologies and interpretation readouts will be required for widespread adoption within the clinic. Machine learning, artificial intelligence, and cohort data pooling, such as the case for positive prediction probability, can present fluorescence imaging as a probability of tumor, rather than an arbitrary signal intensity or contrast value. This further allows the surgeon control in deciding the risk versus reward during surgery, which can be tuned according to the wishes of both the patient and surgeon. Evaluating FMI alongside emerging techniques, such as open-top light sheet microscopy, as well as traditional methods such as serial sectioning histology—augmented by AI techniques to improve histological analysis efficiency—can elucidate their complementary roles. Such comparative analyses are crucial in highlighting the substantial benefits of FMI for comprehensive and timely margin assessments in the treatment of large and complex tumors.

In terms of PDEMA application, despite the time-intensive nature of examining the entire peripheral and deep tumor surface through histopathologic examination of frozen sections, AI-driven image analysis holds promise for significantly shortening frozen-section review times and improving diagnostic completeness in PDEMA—particularly for more complex tumors. Early applications center on relatively straightforward lesions such as BCC and SCC.^6^^,^^89^^–^^91^ For instance, the ArcticAI platform uses graph-based convolutional neural networks to examine multiple tissue sections in parallel, cutting slide reading to as little as 78 s per patient, and remapping tumor findings back to their anatomical origin using inking patterns. Simulated prospective studies have demonstrated how the deployment of these technologies can maximally enhance surgical efficiency and cost-effectiveness. Other emerging AI tools—such as attention-based approaches trained on thousands of slides^92^—can also rapidly localize and identify core tumor areas.

Although WSI enables higher-resolution, cellular-level analysis compared with FMI, it also requires more processing time. One viable workflow to balance speed and detail involves a two-phase approach: first, performing a rapid low-resolution scan to flag suspicious regions, then applying high-resolution imaging to the flagged areas. Advances in graphics^93^ processing now allow for rapid, parallel assessment of tissue sections, including integration of these data into 3D reconstructions at the benchtop. In addition, virtual staining methods, which can simulate permanent section analysis (and even the appearance of immunohistochemical features to guide assessment of more complex tumors),^94^ are emerging as powerful tools to enhance frozen-section quality and more precisely localize tumor cells across a range of tumor types. Notably, AI-enhanced histopathology can also serve as an objective ground truth for developing and validating FMI applications, ultimately improving the calibration of FMI-derived probability maps.

Our proposed method of MAP would include contrast screening at the widefield *en face* margin stage but would utilize cohort data pooling such as positive prediction probability to aid in decision making. Any equivocal tissues that are determined from the microscopic fluorescence imaging stage would progress to digital pathology using the ArcticAI strategies developed by our team that greatly reduce the diagnostic time frame but have high diagnostic value.

## 7 Conclusions

The major bottlenecks to rapid and accurate margin assessment utilizing FMI are similar to those of traditional histopathology-based margin assessment: time for tissue processing, time for image collection, and expertise in interpretation/reading of the images. However, FMI must also consider the state of the tissue, total volume of tissue, and image resolution when considering both the processing time and diagnostic accuracy. A summary of these parameters for imaging surgical margins can be found in Table 1. We propose that MAP will be most successful if used in stages for rapid, low-resolution imaging of manipulated fresh *ex vivo* tissues, and then higher resolution but slower images of fresh and/or frozen sectioned tissues.

**Table 1:** Comparison of imaging strategies in the surgeon-to-pathologist workflow based on a tumor with a 3-cm long axis ($∼75\;{\mathrm{cm}}^{2}$).

Location	Tissue status	Image resolution	Depth of imaging	Total time^a^	Relative diagnostic accuracy
Operating room	*In situ*	Macro	$10^{2}\;\mathrm{mm}$	$10^{0}-10^{2}\;s$	Low
	*Ex vivo*, whole	Macro	$10^{2}\;\mathrm{mm}$	$10^{1}-10^{2}\;s$	Low
Pathology lab	Whole, compressed	Micro	$10^{2}\;\mu m$	$10^{1}\;\min$	Moderate to high
	Cross-sectioned	Meso, Macro	$10^{1}\;\mathrm{mm}$	$10^{1}\;\min$	Moderate
	Frozen sectioned	Micro, Meso	$10^{1}\;\mu m$	$10^{2}\;\min$	High
	FFPE	Micro, Meso	$10^{1}\;\mu m$	$10^{0}-10^{1}$ days	High

a Total time = tissue processing + imaging time.

Several techniques stand out in terms of ease of use and ability to incorporate into a surgery-to-pathology workflow, diagnostic ability, time to diagnosis, and ease of use. Intensity-based imaging is the most cost-efficient, easy to use, and time efficient and has been extensively demonstrated in the standardized workflow during clinical trials. However, intensity-based imaging suffers from being non-quantitative, with intensity values susceptible to variation due to patient/tumor physiology, tissue volume, and optical properties of the sample. This is reflected in the overall low diagnostic accuracies reported by single-agent, intensity-based imaging. Furthermore, surgeon interpretation of the non-quantitative signal of intensity-based FMI requires a steep learning curve and in-depth knowledge of tissue optics and thereby limits the diagnostic accuracy and practical application clinically. Microscopy and lifetime imaging have been demonstrated to produce high diagnostic accuracy but suffer from the need for high technical abilities of the users and substantial equipment costs. Furthermore, the probability that these techniques will be integrated into non-specialized hospitals in the near future remains low. Paired-agent imaging on the other hand utilizes the same, or similar, equipment as intensity-based imaging, is quantitative and fast, and demonstrates high diagnostic accuracies, especially in heterogeneous or low molecular expressing tissues. Integration into clinical workflow would require software updates that allow the calculation of binding potential maps from the fluorescence intensity images and clinical approval of two imaging agents.

**Fig. 9:**
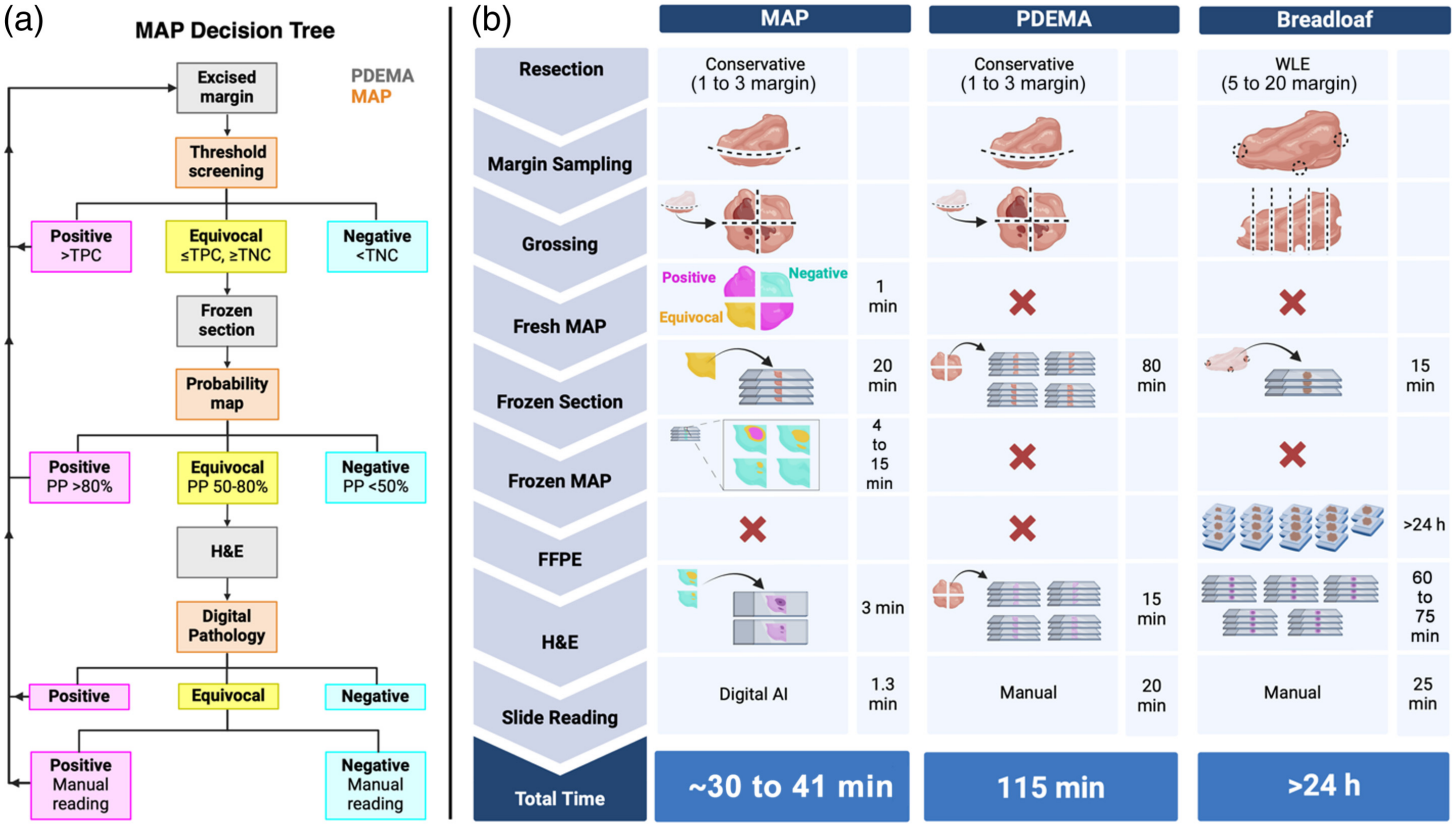
Example of PDEMA margin assessment platform (MAP) digital pathology for a 3-cm (long axis) tumor. (a) MAP decision tree to stratify tissue samples for histological analysis with macroscopic threshold imaging, meso- to microscopic probability mapping, and gold standard confirmation of histology using digital pathology of frozen sections. The key is that only equivocal tissue specimens continue through the pathway at each step, thus reducing the overall analysis time and permitting margin assessment for cases utilizing general anesthesia. (b) Workflow and time-savings afforded by MAP compared with PDEMA and breadloaf analysis in WLE based on a 3-cm long axis tissue. The workflow is based on PAI with initial *ex vivo* images collected on *en face* cut margins using widefield, closed-box imaging. Subsequent PAI images could occur using high-resolution scanning of frozen sections prior to staining and pathological confirmation utilizing digital pathology. TPC: true positive cutoff, TNC: true negative cutoff, PP: positive probability. Created with BioRender.

We propose a MAP methodology using quantitative PAI (Fig. 9), where the resultant binding potential image provides a universal, quantitative metric for diagnosis across patients with high levels of heterogeneity of target receptor expression and can be performed at any tissue stage and image resolution.^75^ The steps to our proposed PAI MAP, as demonstrated in Fig. 9, outline the specimen margin assessment for a 3-cm diameter tumor:

1. **Wide-field imaging of** ***en face*** **margins**. Although preparing *en face* margins requires time, it is the clinical standard of care for many PDEMA methods, such as Mohs surgery. The reduction of the surgical specimen to the *en face* margin increases the contrast of tumor within normal tissues and can be imaged and assessed for maximum PAI signal in under a minute.^72^ We have demonstrated 100% accuracy in stratifying tissues into “tumor” and “normal” based on the maximum PAI signal observed in the tissue; however, we realize the limitation in the small-scale study. We anticipate that rapid assessment of positive tissue would result in the immediate return to the patient to remove an additional tissue layer for assessment. Negative tissue would be fixed and saved for FFPE, the clinical standard. Equivocal tissue would progress to step 2 for further diagnostic stratification. *Estimated tissue preparation, imaging, and diagnostic time:* >1  min. *Diagnostic accuracy:* 90 to 100%.
2. **Mesoscopic imaging of unstained frozen sections**. We have demonstrated that PAI of frozen, unstained tissues with resolutions ranging from 5 to 100 μm using the Odyssey M scanning system is possible with high diagnostic accuracy using positive probability mapping on fresh, frozen sections.^78^ Utilizing cohort data pooling and the positive predictive probability, we anticipate further stratifying tissue sections into positive, equivocal, and negative. Only the equivocal tissues would continue through the MAP protocol, whereas positive would require additional tissue removal from the patient and negative tissues would be sent for FFPE. *Estimated tissue preparation, imaging, and diagnostic time:* ∼24 to 35 min. *Diagnostic accuracy*: 90 to 100%.
3. **Digital pathology**. AI and machine learning have the potential to further enhance the speed and completeness of intraoperative margin assessment by accelerating the reading of equivocal slides identified through PAI for further examination.^6^ Our previously demonstrated ArcticAI platform demonstrated diagnostic assessment and 3D mapping within 78 s per patient with an AUC of 0.97 overall basal cell carcinoma tumor subtypes.^6^ Furthermore, cutaneous squamous cell carcinoma was diagnosed with an AUC of 0.935 at 50-micron resolution.^91^ *Estimated tissue preparation, imaging, and diagnostic time*: ∼4.3  min. *Diagnostic accuracy:* 93 to 97%.

Our proposed MAP demonstrates the utility of incorporating paired-agent fluorescence imaging into the PDEMA workflow. We demonstrate reducing the anticipated processing time of a 3-cm diameter tumor from 115 min with PDEMA alone to an estimated 30 to 41 min using our proposed MAP, which is well within the normal time frame for frozen section processing in solid tumor-wide local excisions. Furthermore, PAI MAP addresses the requirements of quantitative imaging over the micro- to meso-scales, is fast (within the time scale of clinically accepted frozen section analysis), and allows for quantitative digital interpretation. The MAP procedure proposed here is still limited to the scan times and resolutions of commercially available systems. However, these systems are generally intended for a wide array of laboratory work. If PAI MAP were to be clinically integrated, dedicated imaging systems that reduce the data collection lexibility of the general research laboratory systems could be designed, thus making the imaging and diagnostic pathway even more streamlined.

In addition, other groups are also working on similar platforms using confocal microscopy,^88^ autofluorescence FLIM,^38^^,^^43^^,^^87^ and intensity-based imaging.^44^^,^^46^ A multi-stage MAP is a promising method to bring the high diagnostic accuracy and low patient recurrence of PDEMA to solid cancers that require general anesthesia and would not qualify for traditional frozen section methodologies.^3^

## Data Availability

As a perspective article, no code or data were generated.
